# CONE: Community Oriented Network Estimation Is a Versatile Framework for Inferring Population Structure in Large-Scale Sequencing Data

**DOI:** 10.1534/g3.117.300131

**Published:** 2017-08-22

**Authors:** Markku O. Kuismin, Jon Ahlinder, Mikko J. Sillanpӓӓ

**Affiliations:** * Department of Mathematical Sciences, University of Oulu, FI-90014, Finland; † Swedish Defense Research Agency, SE-906 21 Umeå, Sweden; ‡ Biocenter Oulu, FI-90014, Finland

**Keywords:** community detection, graphical models, neighborhood selection, population genetic structure, population graph

## Abstract

Estimation of genetic population structure based on molecular markers is a common task in population genetics and ecology. We apply a generalized linear model with LASSO regularization to infer relationships between individuals and populations from molecular marker data. Specifically, we apply a neighborhood selection algorithm to infer population genetic structure and gene flow between populations. The resulting relationships are used to construct an individual-level population graph. Different network substructures known as *communities* are then dissociated from each other using a community detection algorithm. Inference of population structure using networks combines the good properties of: (i) network theory (broad collection of tools, including aesthetically pleasing visualization), (ii) principal component analysis (dimension reduction together with simple visual inspection), and (iii) model-based methods (*e.g.*, ancestry coefficient estimates). We have named our process CONE (for community oriented network estimation). CONE has fewer restrictions than conventional assignment methods in that properties such as the number of subpopulations need not be fixed before the analysis and the sample may include close relatives or involve uneven sampling. Applying CONE on simulated data sets resulted in more accurate estimates of the true number of subpopulations than model-based methods, and provided comparable ancestry coefficient estimates. Inference of empirical data sets of teosinte single nucleotide polymorphism, bacterial disease outbreak, and the human genome diversity panel illustrate that population structures estimated with CONE are consistent with the earlier findings

Network thinking has opened up new avenues for biological research and has recently gained popularity in fields such as ecology, epidemiology, and population genetics (Proulx *et al.* 2005; Dyer *et al.* 2010; Jombart *et al.* 2011; Dyer 2014; Greenbaum *et al.* 2016). For example, it has improved our ability to understand and disentangle the demographic and evolutionary processes that have shaped the genetic architecture of natural populations and maintained their genetic variation, which has important implications for conservation genetics, medicine, and epidemiological investigations. Here, we show that the concepts of network theory can help to infer interconnections within and between subpopulations, and to better visualize the associated demographic and evolutionary processes.

Recent breakthroughs in high-throughput sequencing technologies have paved the way toward large-scale sequencing of nonmodel organisms. In human genetics, efforts are underway to screen the worldwide genetic variation by sequencing thousands of individuals to generate millions of sequence variants (Abecasis *et al.* 2012). Today, the scope for drawing new conclusions from studies in population genetics is no longer limited by the data-generating process but by the statistical methods available to analyze the vast amounts of data that are generated. In particular, there is a need for new statistical models for handling so-called “large *p*, small *n*” cases, where *p* is the number of markers (parameters) and *n* is the number of individuals sequenced in the population of interest. One promising technique for this purpose is graph modeling, which has several qualities that are advantageous in the analysis of high-dimensional data (Meinshausen and Bühlmann 2006).

Traditionally, distance-based and model-based techniques have been used to explore latent genetic structure at the population level and infer genetic ancestry. Distance-based methods generally rely on multivariate techniques and are also known as model-free or algorithmic methods (Wollstein and Lao 2015). Perhaps the most popular model-free technique is principal component analysis (PCA) (Price *et al.* 2006), which is implemented in software packages such as EIGENSOFT, adegenet, and DAPC (Price *et al.* 2006; Jombart *et al.* 2010). An appealing property of these methods is that they require no assumptions about the underlying population genetic model, and the required computational time is manageable even when analyzing high-dimensional data sets. However, they have some notable drawbacks. First, one must choose a distance metric from a set of many candidates, which determines how the weighting of common and rare alleles in the populations of interest will be set. Second, the lack of a probabilistic framework necessitates the partitioning of individuals via *ad hoc* visualization methods, although alternative solutions do exist (Jombart *et al.* 2010).

Model-based population assignment methods have been used extensively as alternatives to distance-based methods (Pritchard *et al.* 2000; Tang *et al.* 2005; Alexander *et al.* 2009; Frichot *et al.* 2014; Raj *et al.* 2014). The objective of population assignment methods is to use genetic information (molecular markers measured along individual DNA sequences) to assign individuals to subpopulations and provide estimates for the ancestry coefficients. The multinomial-Dirichlet generative model is widely used in popular implementations of this approach such as STRUCTURE (Pritchard *et al.* 2000; Falush *et al.* 2003), TESS (Chen *et al.* 2007), and BAPS (Corander *et al.* 2003). Determining the number of subpopulations is challenging when using these approaches, as is handling large-scale data sets containing thousands of markers. An attempt to overcome these difficulties by using maximum *a posteriori* estimation has recently been published (Raj *et al.* 2014). Here we approach the same problem from a different angle, by estimating the network topology, which reflects the relatedness structure between individuals: We refer to the output (*i.e.*, the construction of a network from individuals) as an individual-level population graph, in contrast to previously reported subpopulation-level works (Dyer and Nason 2004; Dyer *et al.* 2010; Dyer 2014).

An individual-level population graph model in which connections between population members are evaluated based on genetic similarities was recently presented by Greenbaum *et al.* (2016). This approach, like the earlier work of Dyer and Nason (2004) and Dyer *et al.* (2010), used Gaussian models, which might give rise to spurious relationships (Cushman and Landguth 2010). An alternative method using likelihood theory of generalized linear models (GLMs) that can better account for the discrete nature of genotype data and exploit all of the samples in the data set would thus be desirable. Moreover, Greenbaum *et al.* (2016) truncated genetic relationships at a preselected threshold to make their graph sparser and more realistic. Here, regularization techniques such as the LASSO could be appealing alternatives to this approach if paired with a stability selection method (Meinshausen and Bühlmann 2010) to introduce sparseness and find an optimal regularization of the graph in a data-dependent way.

Thus, a novel graph-construction method is proposed that has some important advantages over graph methods which have previously been applied to population genetic data:

1. The population structure problem is divided into smaller components via a neighborhood selection method (Meinshausen and Bühlmann 2006), which enables the analysis of high-dimensional genetic data.
2. The genetic relationship between individuals is exploited to directly determine their connectedness by means of a GLM framework in which the discreteness of multi-locus genotype data are acknowledged.
3. A stability-based subsampling and network modularity-based methods are used to choose the regularization parameter in a data-dependent manner.
4. The number of latent populations does not need to be fixed in the analysis, and by using a suitable community detection algorithm we can better recognize *communities* (*i.e.*, clusters) in the inferred network.
5. Related individuals can be included in the analysis as the method has a natural way of illustrating the relatedness structure.

We have named this multiphase procedure CONE, or “community oriented network estimation.” The CONE process can be divided into five steps: (i) identification of an appropriate model (*e.g.*, LASSO) for the neighborhood selection scheme; (ii) selection of an appropriate tuning parameter for LASSO with, *e.g.*, stability-based subsampling; (iii) analysis of the full data set using the selected neighborhood selection procedure and the tuning parameter determined in the preceding step; (iv) division of individuals into different communities using a suitable community detection algorithm; and (v) estimation of ancestry coefficients.

We examine an undirected graph linked with the exponential family of distributions of random variables. We restrict our focus to genotype data for molecular markers associated with the multinomial distribution, and draw a parallel between the estimated graphical model and the underlying network structure. It is important to note here that our parameterization of multinomial distribution differs clearly from that of assignment methods: we use individual-level genotype probabilities instead of population-level frequency parameters. We also note that while there is a conceptual difference between a *graph* and a *network*, it is hard to make a practical distinction between the two concepts based on their usage in the statistical and genetically oriented literature (see *e.g.*, Dyer and Nason 2004; Meinshausen and Bühlmann 2006, 2010; Friedman *et al.* 2008; Dyer 2014; Greenbaum *et al.* 2016; Li and Jackson 2015). We therefore use the terms network and graph interchangeably.

## Materials and Methods

### Simulated data

We simulated independent microsatellite data sets (two-digit coding) using the software EASYPOP (version 2.0.1) (Balloux 2001). We examined three different scenarios with unequal sample sizes: (i) an island model (scenario 1), (ii) a hierarchical island model with two subpopulations equally grouped into two archipelagos (scenario 2), and (iii) a hierarchical stepping-stone model with two subpopulations separated by a central contact zone (scenario 3).

For each model, 50 data sets were simulated with 10 populations, and (i) a different number of individuals per each population (160 individuals in the first and second population, 80 in the third and fourth, 40 in the fifth and sixth, 20 in the seventh and eighth, and 10 individuals in the ninth and tenth population), and (ii) an even number of individuals (62) for each population. Here, we followed one of the sampling scenarios of Puechmaille (2016). All parameters in EASYPOP were set according to the works of Evanno *et al.* (2005) and Puechmaille (2016): individuals were diploid, with two sexes with an equal number of males and females in each population, and a random mating system was used. Migration rates within sets were set to 0.01 and 0.001 between sets, equal for both sexes. The mutation model was set as a mixed single-step mutation model with an equal probability (0.3) to mutate to any allelic state at a rate of 0.001. Data were generated for a set of 2000 individuals with free recombination between loci, each with two possible allelic states.

The alleles were merged into numerical values 0 (allele pair 0101), 1 (allele pair 0102 or 0201), and 2 (allele pair 0202). Simulations were run for 10,000 generations to ensure equilibrium between drift, migration, and mutation. EASYPOP syntax files, used in the simulations, and the R scripts used for the simulation analysis are provided in Supplemental Material, File S1.

### Simulation of admixed populations

To simulate SNP data from a binomial model, we chose the HapMap phase 3 CEU, CHB, and YRI samples as our ancestral populations. We chose SNPs spaced at least 200 kb apart and the minor allele frequencies calculated over the three populations were used as the true values of the allele-frequency matrix. Then we chose an arbitrary subsample of loci so that the final allele-frequency matrix contained 2500 columns. Finally, we simulated genotypes following the binomial model presented for example in Alexander *et al.* (2009) using six different models for the true values of the *Q* matrix of admixture coefficients. Each simulated data set included 600 unrelated individuals genotyped at 2500 loci. We generated the *Q* matrix independently from the Dirichlet distributions, Dir(β,γ,δ), with different levels of admixture controlled with parameters *β*, *γ*, and *δ*.

### Teosinte data

We initially evaluated the performance of CONE by applying it to a moderately sized SNP genotype data set gathered from 21 different populations of teosinte, the wild ancestor of maize, from various locations in Mexico (Pyhäjärvi *et al.* 2013). The data contains samples from two different subspecies, *Zea mays* subsp. *parviglumis* (11 populations) and *Z. mays* subsp. *mexicana* (10 populations).

Here, we used the same set of markers as Pyhäjärvi *et al.* (2013). We recoded the data with numerical values of 0, 1, or 2, corresponding to the homozygous genotype *AA*, the heterozygous genotype *AB*, and the other homozygous genotype *BB*, respectively. There were no monomorphic markers and SNPs with >10% missing data had previously been removed from the data set. Nevertheless, 16,891 markers had missing sample genotypes: We imputed these missing genotypes (once) with the most common genotype of the corresponding marker (the locus mode). There were also two individuals that did not belong to the wild maize populations and were identified as maize hybrids within the *mexicana* samples. These hybrids were not identified in the public data set so we did not remove them from our data; we expected that the corresponding individuals would have insignificant effects on our population structure analysis. Our final data set thus contained 250 samples (columns *N*) and 36,719 markers (rows *L*) with no missing genotypes. We also performed some fine-scale analysis over the *parviglumis* and *mexicana* subspecies. For these data subsets, we again used the same 36,719 SNPs as Pyhäjärvi *et al.* (2013), but due to the smaller sample sizes, we removed the monomorphic markers. Imputation was performed as described above. The final data set for *parviglumis* populations contained 130 samples and 35,727 SNPs, while the *mexicana* data set contained 120 samples and 35,500 SNPs. The teosinte SNP data set together with some supplemental files are available at http://datadryad.org/resource/doi:10.5061/dryad.8m648.

### Escherichia coli data

Salipante *et al.* (2015) analyzed 312 blood- and urine-derived isolates of extraintestinal pathogenic *E. coli* (ExPEC), a common agent of sepsis and urinary tract infections. The isolates were obtained during the course of routine clinical care at a single institution. Whole genome analysis was performed to infer phylogenetic relationships and detect partitions in the population. A subset of 120 genomes was selected for analysis here. Draft genomes were aligned against the reference *E. coli* K12MG1665 genome (GenBank ID: 556503834) using ProgressiveMauve (Darling *et al.* 2010) with default settings. A phylogenetic tree was obtained using the neighbor-joining algorithm in MEGA6 (Tamura *et al.* 2013).

The data set contained 121 strains, including the reference K12MG1665 and 348,859 SNP markers. Before conducting our analysis, we removed SNPs with >5% missing alleles. We then imputed the remaining missing genotypes (once) with the most common variant at the corresponding site. The final data set contained 333,549 SNPs with no monomorphic markers.

We recoded the data with numerical values of 1, 2, 3, and 4 according to the allele (*i.e.*, nucleotide) present at each locus. Because the data set pertains to a haploid organism, we note that in contrast to Equation 1 there are now four vectors to consider ${\hat{\beta }}_{g},$ g=1,…,4, corresponding to each individual locus instead of allele pairs. Using numerical values 1, 2, 3, and 4 does not violate our neighborhood selection framework because CONE can be generalized to an arbitrary number of genotypes (classes). CONE can thus be implemented exactly as described later in the article: an estimated edge exists between nodes (strains) *i* and *j*, i,j=1,…,121, if and only if ${\hat{\beta }}_{g}$ is nonzero for some *g*.

### HGDP data

We used a SNP data set, named the Stanford HGDP SNP genotyping data set (Rosenberg *et al.* 2002). The data set contains 1043 individuals from 51 different populations and 660,918 SNP markers. We recoded the data with numerical values of 0, 1, or 2 according to the number of reference alleles C or G in the genotype. As was also done by Raj *et al.* (2014), we removed SNPs that were monomorphic or had >5% of the genotypes missing. We then imputed the missing genotypes (once) with the most common genotype of the corresponding marker, as was done with the teosinte data. The final data set included 659,421 markers and 1043 individuals. In contrast to the approach adopted by Li *et al.* (2008) and Raj *et al.* (2014), we did not modify the data prior to analysis by performing a Hardy–Weinberg equilibrium test or removing related individuals; this did not adversely affect the CONE procedure and the results obtained were comparable to those reported previously (Li *et al.* 2008; Raj *et al.* 2014). The data are freely available at http://www.hagsc.org/hgdp/files.html and are also deposited at Fondation Jean Dausset-CEPH (http://www.cephb.fr/en/hgdp_panel.php#basedonnees).

### Graphical model

Let $X={\left(X_{1},\ldots ,X_{N}\right)}^{\text{T}}$ be a random vector of *N* individuals. The undirected graph can be used to visualize the relationships between these individuals. We describe such a graph as a population graph because it can be used to illustrate the individual-level population structure in a similar way to software such as STRUCTURE, without requiring any prior information about the population structure. Let G=(N,ε) be a graphical model, where N={1,…,N} is a set of nodes and ε is a set of edges (i,j), i,j=1,…,N, i≠j in N. Each individual corresponds to one node in the set N and a pair (i,j) is contained in the edge set ε if and only if individual $X_{i}$ is conditionally dependent on individual $X_{j}$ given all remaining individuals. More informally, there is a probabilistic relationship between individuals $X_{i}$ and $X_{j}$ when the effect of all remaining variables is eliminated; if this condition is satisfied, we say that there is an edge between *i* and *j* or vice versa.

With large data sets containing thousands of individuals and enormous numbers of SNP markers, solving the graph-selection problem quickly becomes unfeasible. However, the problem can be made simpler by adopting a regression approach whereby one estimates the neighborhood of the node *i* by performing a regression of the variable $X_{i}$ on the rest of the variables. The main motivation for using regression in network analysis is to divide the graph-selection problem into much smaller so-called “neighborhood selection problems” (Meinshausen and Bühlmann 2006). Analyzing each node one by one makes it possible to analyze large data sets by estimating *N* nodes, each of size $2^{N-1}.$ Dividing the graph construction into smaller subproblems also makes it possible to use parallel computing to increase time efficiency. Graph estimates obtained using the strategy outlined above can be described as dependency networks (Heckerman *et al.* 2000).

In this discussion we ignore some of the more sophisticated issues relating to neighborhood selection; we refer the reader to publications such as that of Meinshausen and Bühlmann (2006) for information on graphical models associated with the Gaussian distribution. We approach the neighborhood selection as a regularized linear regression problem (*i.e.*, a problem of penalized regression) and use an N×N symmetric adjacency matrix to depict the relationships between individuals (nodes). The elements of the adjacency matrix are either zero or one. Each zero entry of the matrix indicates absence of an edge between the nodes represented on the corresponding column and row. For convenience, we will refer to this kind of neighborhood selection procedure based on *L*_1_-penalized (a so-called LASSO penalty) regression as Meinshausen and Bühlmann-style (MB-style) neighborhood selection.

In the case of undirected graphs, the relationships between nodes are visualized by drawing undirected lines (edges) between related nodes. With Gaussian data, conditional independence is related to partial correlations, and is consistent with the examination of zero entries in the Gaussian inverse covariance matrix (Edwards 2000). Here we are more interested in neighborhood selection, which is a subproblem of covariance selection for Gaussian data.

### The neighborhood selection scheme

For diploid organisms, we assume that each locus in the population has *P* possible genotypes with nonzero frequencies. In general, we can assume that P>2 and the categories have no specific order. For every sample (individual) there are now *P* different random variables corresponding to the different genotypes, and the molecular markers for each sample are divided into these categories. For one locus of a diploid organism, there would be three categories corresponding to three different genotypes *AA*, *AB*, and *BB*, meaning that P=3. For the sake of simplicity, we will describe our method for individuals representing diploid organisms. Thus, each individual determines multiple different random variables and their joint probability distribution through a multinomial distribution ${\text{Multinomial}}_{i}\left(p_{1},p_{2},p_{3};L\right),$ where $p_{1},p_{2}$ and $p_{3}=1-p_{1}-p_{2}$ correspond to the genotype probabilities of the *L* measured markers of individual *i*, i=1,…,N. Again, $p_{1},\;p_{2},$ and $p_{3}$ have nothing to do with population frequencies. However, it is natural to expect that populations have shared genotype frequencies and, thus, individuals from the same population group have similar genotype probabilities. We also assume that the random variables (genotypes) of two related population groups affect the parameters (genotype probabilities) of both groups; this corresponds to gene flow, with no specific direction, between population groups and is expressed by the presence of undirected edges linking different individuals in the graphical model.

In the original work of Meinshausen and Bühlmann (2006), only the properties arising from Gaussian data were examined. We apply MB-style neighborhood selection to SNP (genotype) data measured from either haploid or diploid organisms; different loci are seen as independent, replicated observations from the same model (linkage equilibrium). The LASSO method is applied in the usual way for Gaussian distributed variables, and coefficient estimates are obtained by minimization of the regularized mean squared error (Tibshirani 1996). With GLMs, the negative log-likelihood is minimized instead.

We consider a multinomial model for the categorical responses (see, *e.g.*, Hastie *et al.* 2009, pp. 120–122). The data consist of a L×N matrix $X=\left(X_{1},\ldots ,X_{N}\right),$ and each vector $X_{i}={\left(X_{i1},\ldots ,X_{iL}\right)}^{\text{T}},$ i=1,…N can contain at most three genotypes per locus. We focus on one individual $X_{i}$ and denote the remaining marker data by $X_{-i}=X\backslash \left\{X_{i}\right\}.$ The procedures described in the remainder of this section are then applied to each individual, one by one. Stated formally, our objective is to answer the question: “How does the distribution of genotypes of individuals $X_{j},$ j=1,…,i−1,i+1,…,N explain the genotype of the individual *X_i_*?”

The basis of our analysis is the multi-logit model (Friedman *et al.* 2010)

$$\mathrm{Pr}\left(X_{il}=g|X_{-i}\right)=\frac{\exp\left\{\beta_{0g}+x_{-i}^{\text{T}}\beta_{g}\right\}}{\sum_{k=1}^{P}\exp\left\{\beta_{0k}+x_{-i}^{\text{T}}\beta_{k}\right\}},$$ (1)

in which $\beta_{g}$ is a vector of dimension N−1 and g=1,2,3. For simplicity, let $p_{g_{l}}\left(x_{-i}\right)=\mathrm{Pr}\left(X_{il}=g_{l}|X_{-i}\right),$ where $g_{l}=\left\{1,2,3\right\}$ corresponds to the *g*th genotype for the *i*th individual at the locus *l*, l=1,…,L.

The negative log-likelihood for the individual $X_{i}$ is

$$-l\left(\beta_{g};\text{data}\right)=-\sum_{l=1}^{L}\log p_{g_{l}}\left(x_{-i}\right).$$ (2)

We minimize the LASSO-style penalized negative log-likelihood

$$\arg\underset{\beta_{g}}{\min}\left[-l\left(\beta_{g};\text{data}\right)/\text{L}+\lambda {\left\|\beta_{g}\right\|}_{1}\right],$$ (3)

where ${\left\|\beta_{g}\right\|}_{1}$ is the *L*_1_-norm of a vector $\beta_{g}$ and *λ* is the so-called tuning parameter, λ≥0. It is well known (see for example Tibshirani 1996) that in such cases, $L_{1}$ regularization will yield vectors ${\hat{\beta }}_{g},$ with s<N−1 nonzero elements depending on the value of *λ*.

When dealing with multinomial regression, we obtain three sparse vectors whose elements differ from each other as estimates of $\beta_{g}$ for each genotype category *g*. If performing a standard LASSO regression for Gaussian data, we would construct a graph, as described by Meinshausen and Bühlmann (2006), by noting that there is an undirected edge between nodes *i* and *j*, j=1,…,i−1,i+1,…,N if and only if $X_{i}$ and $X_{j}$ are conditionally dependent given all other individuals. Thus, in the LASSO setting for Gaussian data, there is no edge between *i* and *j* if ${\hat{\beta }}_{j}=0.$

With our multinomial setting we must use a different guideline to determine the dependency between individuals. We propose an intuitive rule to determine whether or not there is an indication of dependency between different individuals: set an estimated edge between *i* and *j* if and only if ${\hat{\beta }}_{g}$ is nonzero for some *g*. This corresponds to a value of one in the *j*th row of the *i*th column of the adjacency matrix.

Because the probability $p_{g_{l}}\left(x_{-j}\right)$ also produces a dependency structure between individuals $X_{j}$ and $X_{i},$ we use the so-called “and” rule to ensure that the adjacency matrix is symmetric: there is an edge between the nodes *i* and *j* if and only if “*X_i_* depends on *X_j_*” *and* “*X_j_* depends on *X_i_*” given all other individuals. If the rule holds, the adjacency matrix has one as an entry on the *i*th row and *j*th column, and vice versa. Otherwise, the entry is zero. Alternatively, one could use a so-called “or” rule that states that there is an edge between the nodes *i* and *j* if and only if “*X_i_* depends on *X_j_*” *or* “*X_j_* depends on *X_i_*” given all other individuals.

The multinomial model cannot be used for neighborhood selection if one of the (three) possible genotypes has a very low or zero frequency over all loci of the samples. However, the probability of this happening with large marker data sets is practically zero.

The advantage of the penalized multinomial model is that none of the individuals are forced to be part of some population, unlike in analyses performed with model-based methods. The neighborhood selection method can be used to identify these individuals and they are represented by free-floating nodes with no edges connecting them to other nodes. No predetermination of the number of subpopulations is needed because we assume that the method will naturally divide the individuals into different populations. By itself, constructing a network estimate is not sufficient to produce easily interpreted results; we still need to separate each cluster as a separate connected component, *i.e.*, a cluster of nodes with few or no edges linking them to other clusters. Dividing nodes (individuals) into clusters is discussed in the *Detecting communities* subsection.

One can use either frequentist or Bayesian methods in the analysis. We used the R package glmnet (version 2.0-2) to fit the regularized GLMs. The glmnet package exploits the cyclic coordinate descent algorithm, which has proven to be a fast method for solving LASSO-type problems such as Equation 3, and can work on very large data sets. For more details about the glmnet package, see Friedman *et al.* (2010). Because R stores all objects in memory, analyzing large data sets becomes practically impossible even though the glmnet package can handle very large vectors and make good use of sparse data. We therefore used R packages such as bigmemory (version 4.5.10) and ff (version 2.2–13) to handle multi-gigabyte data sets and variables in R. Packages bigmemory and ff can store objects to the hard drive while preserving R’s strong capacity for efficient statistical analysis.

### Choosing the tuning parameter

The neighborhood selection scheme is comparatively easy to use and implement as nice statistical properties are obtained by adjusting a single penalty parameter *λ* (Raskutti *et al.* 2009; Liu *et al.* 2010). Various common model selection methods could be used to identify the optimal value of the tuning parameter, including k-fold cross-validation, Akaike’s information criterion, or the Bayesian information criterion (see, *e.g.*, Hastie *et al.* 2009, pp. 230–245). In practice it is well known that cross-validation and information criteria are often impractical in graph construction because their application usually produces rather dense graphs with many false-positive edges (Wasserman and Roeder 2009; Liu *et al.* 2010).

In this article we represent two methods that can be used to choose a proper value for the tuning parameter, one using a stability approach and the other, modularity approach, which is similar to the so-called “elbow” method, commonly applied in hierarchical clustering (see *e.g.*, Zhang and Horvath 2005; Zumel and Mount 2014, pp. 187–190).

#### Finding the optimal tuning parameter—The stability approach:

Liu *et al.* (2010) used a stability approach to regularization selection (StARS) to choose the most stable graph. The StARS is a general way of obtaining an undirected graph-selection criterion when the graph estimation is based on the penalized likelihood minimization problem as in Equation 3. StARS guarantees that the graph estimate will be consistent, provided that some moderate conditions are met (see theorem 4.2 in Liu *et al.* 2010). Our regularization selection procedure is based strictly on the methods described in the work of Liu *et al.* (2010).

**Figure 1:**
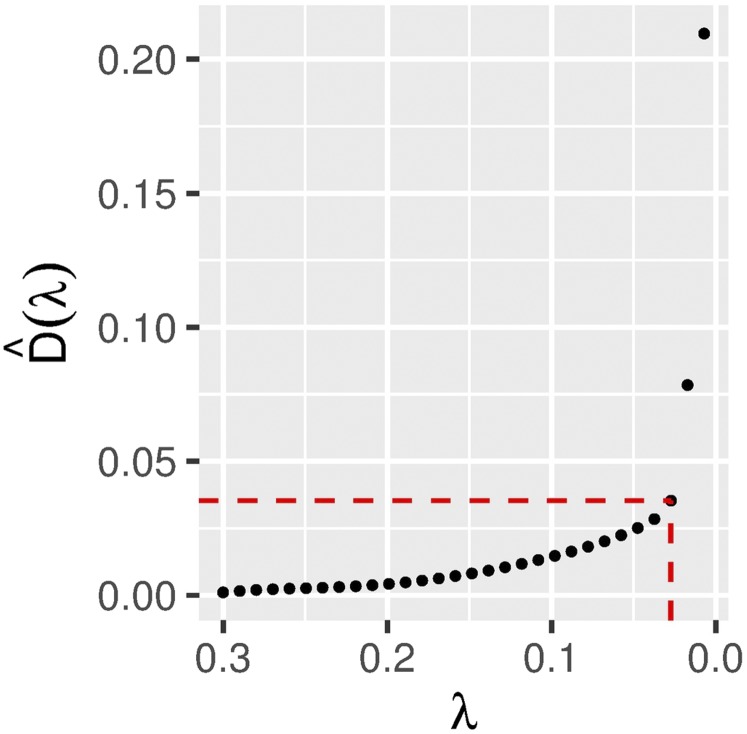
The StARS instability measure plotted against the tuning parameter. The red dashed line indicates the value smaller than or equal to the threshold value (β=0.05) on the vertical axis, and the corresponding value of the tuning parameter on the horizontal axis. The simulation model considered here is the island model described in subsection *Simulated data*.

Suppose we want to choose the “best” value for the tuning parameter *λ* in Equation 3 over a sequence of different positive values. With StARS, we use subsampling to measure the uncertainty regarding the presence of edges between the nodes in the graph estimate $\hat{G}\left(\lambda \right)$ for each fixed value of *λ*. Then we calculate the total instability of the estimated graph based on the subsamples. Finally, we choose an estimate $\hat{\lambda }$ for *λ* that gives the optimal stability among the sparse graphs based on a special instability measure $\hat{D}\left(\lambda \right)$ associated with the corresponding graph estimate. Assuming the sequence of the instability measure is monotonic, we can choose $\hat{\lambda }=\min\left\{\lambda :\hat{D}\left(\lambda \right)\leq \beta \right\}$ with a threshold parameter *β*. We use β=0.05 as a default value for our StARS procedure, as proposed in Liu *et al.* (2010). The selection of *λ* using the quantity $\hat{D}\left(\lambda \right)$ is illustrated for the case of a data set in Figure 1.

Although the StARS analysis is computationally intensive, we favor it because it is readily interpreted and efficiently reduces the number of false-positive edges. It also provides an additional graph estimate based on the subsamples of size *b*, which can be used to analyze the connectivity of the estimated graph as described above to determine how the subpopulations are differentiated from each other. We present some guidelines for reducing the computation time needed for graph estimation. In principle, the tuning parameter *λ* should be varied from zero to a moderately large value. However, the use of small values will slow down the glmnet package despite its ability to time efficiently produce the whole solution path. In practice, the graph will be overly dense if relatively small values of *λ* are used, so it is reasonable to impose a lower bound above zero on this parameter. Additionally, the number of subsamples should be quite large in principle: because the processing time is directly proportional to the number of subsamples, using many subsamples would greatly increase the time needed to execute the StARS procedure. However, our analysis results suggested that even relatively small numbers of subsamples yielded reasonable results. We use M=20 subsamples for our StARS selection procedure, which is also the default value for selecting the most stable tuning parameter for the graphical lasso (glasso) algorithm (Friedman *et al.* 2008) in StARS analyses performed with the R package huge (version 1.2.7) (Zhao *et al.* 2012). Taking that into account, under our experimental conditions, the cyclic coordinate descent algorithm used in the glmnet package may fail to converge for small values of the tuning parameter *λ*. This usually occurred when working with small data sets using the StARS procedure. This problem could be circumvented by tailoring the grid for the problematic candidate *λ* values or generating a new subsample. Problems of this kind were not encountered when working with real-world data sets here. Before we can describe how the elbow method can be used to choose a proper value for the tuning parameter or how CONE can be used for estimation of ancestry coefficients, we have to first explain some concepts in network *communities*.

#### Detecting communities:

The word “subpopulation” is used to refer to the known grouping of individuals and the words “cluster” or “community” to refer to the grouping of individuals inferred in the data set with the method described below. We do not assume that different subpopulations usually form groups that will be totally separate from all other subpopulations; rather, we assume that only very genetically distant individuals will not be directly joined together. However, it is natural to presume that the connections between more distantly related groups will be much weaker than those between closely related groups; namely, individuals from the same subpopulations will form different *communities* in the network. The definition of a *community* or *cluster* in network theory is quite loose; a community is a group of nodes having a relatively large number of interconnecting edges but looser connections to other groups or nodes in the network (Fortunato 2010).

Although the neighborhood selection procedure generates a network estimate that includes estimates of the strengths of the edges between different nodes, we must use some external algorithm to distinguish communities from one another. In a similar way to Greenbaum *et al.* (2016), we use a community detection algorithm for this purpose. Specifically, we used the R packages qgraph (version 1.3.2) (Epskamp *et al.* 2012) and igraph (version 1.0.1) to draw undirected graphs based on the adjacency matrices, and we used the Fruchterman–Reingold algorithm (Fruchterman and Reingold 1991) and the Walktrap algorithm (Pons and Latapy 2006) as implemented in these packages for community partitioning.

The basic principle of the Fruchterman–Reingold algorithm is that each node repulses the others, but nodes (individuals) that are connected by edges are drawn closer together: This gives rise to a force-directed layout that is easier to interpret than the original graph, with a minimal number of crossed edges and more or less separate communities. The principle behind the Walktrap algorithm is that nodes belonging to the same community have more edges connecting each node to another. The *random walks* on a graph tend to get stuck into these “dense” parts (communities) of the graph. Thus one can compute a distance measurement of the structural similarity between nodes and between communities, a *modularity* measure. Finally, a hierarchical clustering algorithm is used to detect different communities in the graph, and the partition that maximizes the modularity measure is chosen as the layout with different nodes grouped to more or less distinct communities.

Here our work differs from that of Greenbaum *et al.* (2016), who used the Girvan–Newman algorithm (Girvan and Newman 2002) in their main analysis (however, their supplemental materials include a discussion on the use of alternative community detection algorithms for network construction, including the Walktrap algorithm). Importantly, in the case of the additional StARS graph, the Fruchterman–Reingold algorithm can distinguish between nodes to separate communities in a way that facilitates interpretation. While alternative community detection algorithms are available, we have found Walktrap and Fruchterman–Reingold algorithms to give reasonable and consistent results.

Puechmaille (2016) noticed that uneven subsampling affects STRUCTURE’s ability to infer the number of subpopulations from a data set. Specifically, small populations tend to separate into different subpopulations in STRUCTURE analyses. We did not encounter this problem with CONE in our real data analyses, although its community detection algorithms usually give the best results when applied to evenly distributed and sufficiently large communities. Moreover, the comparatively simple community layout produced by CONE analyses makes it possible to identify possible estimation errors by visual inspection.

For more detailed descriptions of community analysis and reviews of the various community detection algorithms, see for example Fortunato (2010), Lancichinetti and Fortunato (2009), and Harenberg *et al.* (2014).

#### *Finding the optimal tuning parameter*—The network modularity approach:

In population structure analysis it is a common task to (pre)determine the number of populations in the data set when using model-based methods to derive the ancestry proportions from the data (Pritchard *et al.* 2000; Alexander *et al.* 2009; Frichot *et al.* 2014; Raj *et al.* 2014). Although somewhat of an artificial concept, one has to choose the number of populations (*K*) for the analysis, even if in practice there will be no “true” value of *K*, as samples from real populations rarely conform to the assumptions of the model.

With CONE, one does not need to predetermine the number of populations. Nevertheless, it is useful to have knowledge about the number of clusters to help understanding of the (visual) appearance in the data. In particular, inferring the number of populations is useful if CONE is used for estimation of ancestry coefficients. In such cases, one can learn *K* directly from the data using methods common to hierarchical clustering (see, *e.g.*, Zhang and Horvath 2005). For this purpose we use the Walktrap algorithm for community detection, considering that the hierarchical clustering algorithm is a part of the Walktrap algorithm. Similar to hierarchical clustering methods, we derive network estimates with various values of the tuning parameter *λ*, determine *K* to be equal to the number of communities found with the Walktrap algorithm, and network modularity equal to that of the given grouping. We note that the modularity value determined by the Walktrap varies in between −1 and 1. A positive modularity value indicates a better separation between communities of the graph. After inferring the number of clusters and network modularity with different values of the tuning parameter, we examine if there is an elbow in the network modularity *vs.* the number of inferred cluster plots. We observed that the number of inferred communities seem to stabilize similarly to the hierarchical cluster analysis.

**Figure 2:**
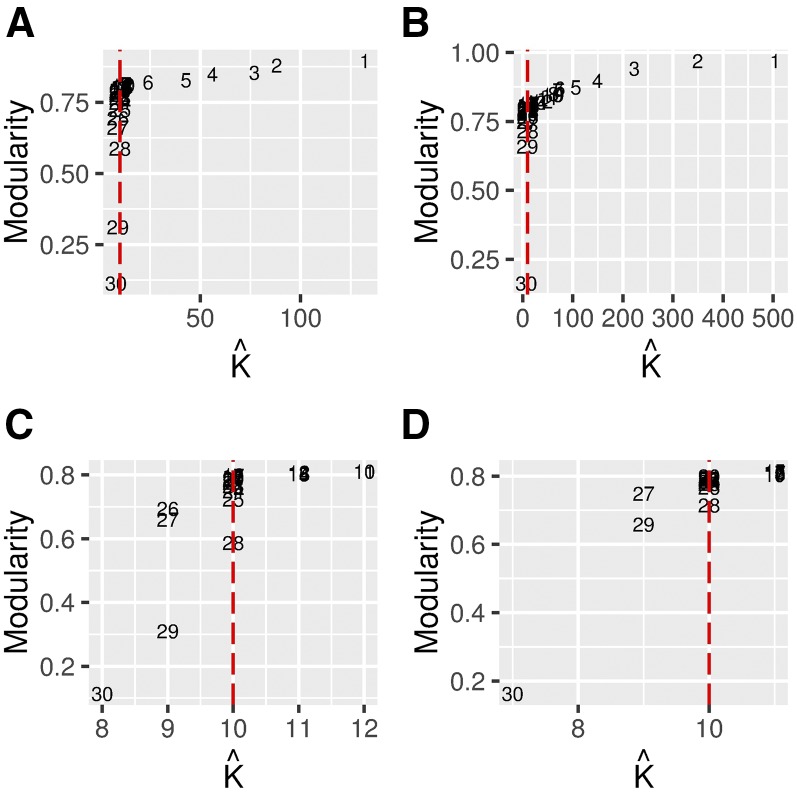
The network modularity corresponding to different numbers of inferred communities ($\hat{K}$) with different values of the tuning parameter. (A) Networks computed with subsampling. (B) Unweighted networks inferred with MB approximation. (C) Scaled (A). (D) Scaled (B). The red horizontal dashed line indicates the true number of subpopulations (K=10). Each number in the plot area corresponds to an index number *i* ($\lambda_{i}$) for different ascending values for the tuning parameter. The simulation model considered here is the island model described in subsection *Simulated data*.

We have illustrated the procedure described above in Figure 2. The optimal value for the tuning parameter is a trade-off between the network modularity and sparseness. In Figure 2, there is a clear elbow around the 17th and 27th value of *λ*. Here, the elbow is quite unambiguous but this may not always be the case; in Figure 2C the subsampling of the data causes small instability in the procedure. One could also use a slightly smaller value for *λ* but “too small” values should be avoided because they will lead to low network modularity and network estimates with several insignificant edges (connections) between nodes (individuals).

### Computing ancestry coefficients

Estimation of ancestry coefficients is an essential part of model-based methods. Considering how model-based methods derive the relationship between ancestry proportions and an individual’s alleles, degree of ancestry can be directly estimated from model parameters. In this section, we use the term “ancestry coefficients” to refer to the estimated ancestry in general. Denote so-called ancestry coefficient matrix as $Q=\left(q_{ik}\right),$ where $q_{ik}$ represents the fraction of individual *i*’s genome originating from the ancestral population *k*. The matrix *Q* has dimension N×K. An obvious drawback of visual inspection methods such as PCA and the network analysis is that they do not directly produce an estimate for the ancestry coefficient matrix *Q*, although there is a way to estimate *Q* with PCA using the DAPC program (Jombart *et al.* 2010).

Considering that elements in *Q* represent ancestral proportions of individuals in *K* possible subpopulations, there seems to be a natural connection between ancestral proportions and network estimates; the edges in the network computed with the neighborhood selection algorithm represent the genetic relationship between different individuals in the network. It is consistent to assume that the proportion of edges of a node (representing an individual) between other nodes belonging to a community represents the fraction of genome from the corresponding ancestral population. Thus one can gain an estimate of *Q* with CONE using the method described below.

Assume that the nodes N in the graphical model G=(N,ε) are partitioned in *K* distinct communities, $N=\left\{C_{1},\ldots ,C_{K}\right\}$ and $A=\left(a_{ji}\right)$ is the symmetric N×N adjacency matrix which corresponds to the estimated network. Element $a_{ji}$ can be either all zeros and ones, corresponding to an unweighted network, or can vary between zero and one, representing the weighted network. Diagonal elements of *A*, $a_{ii}$ are set to zero. The degree of a node *i* denoted by $D_{i}$ can be computed as the column sum of the *i*th column of *A*, $D_{i}=\sum_{j=1}^{N}a_{ji}.$ Clearly $0\leq D_{i}\leq N-1$ for all i=1,…,N. We determine the estimate of the ancestry proportion of individual *i* in the ancestral population *k* as the proportion of the number of edges belonging to the community *K* and the overall node degree, ${\hat{q}}_{ik}=C_{k}/D_{i};$ where $C_{k}$ is the number of edges of node *i* which are connected to other nodes belonging to the community $C_{k},$ $C_{k}=\sum_{j=1}^{N}I_{k}a_{ji};$ where $I_{k}$ is an indicator variable that takes the value one if the node *j* belongs to a community $C_{k}$ and zero otherwise, and $1\leq C_{k}\leq N-1,$ k=1,…,K.

If $D_{i}$ takes the value zero, the corresponding ancestry proportion estimate $q_{ik}$ is set to one, meaning that this particular individual is not genetically related to any other individual in the sample and comes from some spurious cluster that has not been removed by the neighborhood selection method. Similarly to the model-based assignment methods, such as STRUCTURE, our approach suffers from a label-switching problem, *i.e.*, it is not invariant to the permutations in the terms of different communities and can alter with different genotype matrices with the same underlying ancestral populations. Solving the label-switching problem is an open research question but some solutions have been presented (see, *e.g.*, CLUMPP software; Jakobsson and Rosenberg 2007).

### Genomic relationship matrix

The genomic relationship matrix, as described by VanRaden (2008), $K=\left(K_{ij}\right),$ i,j=1,…,N is obtained as follows: $K=Z^{\text{T}}Z/\left(2\sum p_{l}\left(1-p_{l}\right)\right),$ where *Z* is the matrix with mean allele effects set to zero. This in turn is obtained by subtracting *P* from *M*, where *P* is a 1×L column matrix with column elements $2\left(p_{l}-0.5\right),$ and $p_{l}$ is the frequency of the reference allele at locus *l*, l=1,…,L. Matrix *Z* is commensurate with our data matrix *X* after recoding to −1, 0, and 1 into matrix *M*. We define a pair of strongly genetically related individuals $X_{a}$ and $X_{b}$ as any pair for which the corresponding element of *K* is >0.5, that is $K_{ab}>0.5.$

### Data availability

The authors state that all data necessary for confirming the conclusions presented in the article are represented fully within the article. EASYPOP software is available from http://www.unil.ch/dee/en/home/menuinst/softwares--dataset/softwares/easypop.html. 

All SNP genotypes and related information for *Z. mays* subsp. *parviglumis*, *Z*. *mays* subsp. *mexicana*, and *Tripsacum dactyloides* files are available from the DRYAD database (http://dx.doi.org/10.5061/dryad.8m648).

*E. coli* draft genomes are publically available at NCBI GenBank (http://www.ncbi.nlm.nih.gov/genbank) under accession numbers JSFQ00000000–JSST00000000. The reference strain K12 substrain MG1665 has the GenBank ID 556503834.

All Stanford HGDP SNP genotyping data files are deposited at Fondation Jean Dausset-CEPH (http://www.cephb.fr/en/hgdp_panel.php#basedonnees).

## Results

### Estimating the number of clusters in simulated data

To illustrate how CONE is able to estimate the true number of (sub)populations, we simulated SNP data under three different models of population structure: the island model (scenario 1), hierarchical island model (scenario 2), and hierarchical stepping-stone model (scenario 3). Overall there were 10 subpopulations in each migration model. In particular, we demonstrate how uneven sample sizes do not have a serious effect on CONE’s ability to detect the correct number of clusters (inferred subpopulations).

We determined the number of inferred clusters $\hat{K}$ with CONE and sNMF (Frichot *et al.* 2014), where the latter represents model-based methods. sNMF was run using the R package LEA (version 1.6.0). We used the Walktrap community detection algorithm implemented in the R’s igraph package to infer the number of different communities in our graph estimate. For sNMF we chose the optimal number of clusters which minimized the cross-entropy criterion (Frichot *et al.* 2014). We ran sNMF with the number of populations changing from 1 to 15. We calculated five runs for each *K* value in each scenario to gain a better estimate for the cross-entropy criterion. The nonnegative regularization parameter *α* was set to 100 with sNMF. The choices we have made are used only for illustrative purposes and both CONE and sNMF can be used in a more adaptable manner. The performance of the STRUCTURE program has been studied extensively (Puechmaille 2016; Evanno *et al.* 2005) with multiple different sample sizes and sampling strategies; the program STRUCTURE using ΔK statistics (Evanno *et al.* 2005) either detects the uppermost hierarchical level of population structure (even sample sizes) or fails to detect both the upper or lower hierarchical levels of population structure (uneven sample sizes).

With CONE, we used the network modularity approach and StARS to choose the optimal value for the tuning parameter to estimate *K*. For illustrative purposes only and to avoid unnecessarily long-lasting simulation loops, we used just one of the replicated data sets as a validation set with the modularity approach and chose the value of *λ* according to the analysis of the validation data set. Then, we continued the simulation analysis using the value of *λ* determined from the validation set for each of the 50 replicates. We used the root-mean-square error $\text{RMSE}\left({\hat{K}}_{i},K\right)={\left[\sum_{i=1}^{50}{\left({\hat{K}}_{i}-K\right)}^{2}/50\right]}^{1/2},$ where ${\hat{K}}_{i}$ corresponds to an inferred number of clusters out of 50 estimates, as the error measure. Results are reported in Table 1.

**Table 1:** Summary of the simulation results when detecting the true number of subpopulations ***K*** (uneven sample sizes)

Method*^a^*	Scenario 1	Scenario 2	Scenario 3
CONE (Mod)	10 92% (0.28) 9 **11**	10 82% (0.49) 9 **12**	10 82% (0.42) 9 **11**
CONE (StARS)	10 52% (1.00) 7 **11**	10 82% (0.42) 9 **10**	10 62% (0.71) 8 **11**
sNMF (CE)	10 48% (1.17) 9 **13**	11 44% (1.56) 10 **14**	12 18% (2.77) 9 **15**
CONE (subsample)	9 26% (1.57) 7 **11**	10 82% (0.55) 8 **10**	11 46% (1.38) 10 **13**

Island model (scenario 1), hierarchical island model (scenario 2), and hierarchical stepping-stone model (scenario 3). Mod, modularity; CE, cross-entropy.

a We have reported the median, percentage of the number of correctly inferred subpopulations, an error measure (RMSE), and minimum and maximum (in boldface) values of the number of inferred subpopulations. We used both the modularity approach and StARS to choose a proper value for the tuning parameter *λ*. We have also reported the number of inferred clusters identified with the StARS subsampling (subsample).

CONE, using the Walktrap community detection algorithm, outperformed the cross-entropy criterion used with sNMF in all scenarios when the tuning parameter is chosen based on the network modularity or StARS. When the tuning parameter is chosen via StARS using the default value 0.05 for *β*, the number of subpopulations is somewhat underestimated in all scenarios. Overall, CONE seems to detect the lower hierarchical level of structure in all scenarios. In an additional analysis, we examined how the additional weighted graph computed as a part of the StARS procedure was able to distinguish different communities from each other; these estimates are summarized in the last row in Table 1, named as CONE (subsample). In both island models, communities detected from the weighted graph seem to produce downward-biased estimates of the true number of subpopulations. On the other hand, in the hierarchical stepping-stone model, the weighted graph overestimated the true number of subpopulations. The results obtained with sNMF, using the cross-entropy criterion, suffered from uneven sample sizes but, in contrast to the earlier findings (Puechmaille 2016), it did not underestimate the true number of subpopulations. That is, sNMF was able to detect the lower level hierarchical structure and never detected the upper hierarchical level of structure in the hierarchical models simulated. In the island model, sNMF gave even more accurate estimates of the true number of subpopulations compared to the corresponding estimates inferred from the weighted network. To comprehensively illustrate that the bias in estimated number of subpopulations is due to uneven sampling, we repeated the simulation analysis using even samples. As expected, both CONE and, in particular, sNMF were able to produce more accurate estimates of the true number of subpopulations when the sample sizes were even (Table 2).

**Table 2:** Summary of the simulation results when detecting the true number of subpopulations ***K*** (even sample sizes)

Method*^a^*	Scenario 1	Scenario 2	Scenario 3
CONE (Mod)	10 96% (0.20) 10 **11**	10 98% (0.14) 10 **11**	10 94% (0.24) 10 **11**
CONE (StARS)	10 100% (0.00) 10 **10**	10 100% (0.00) 10 **10**	10 92% (0.37) 8 **10**
sNMF (CE)	10 68% (0.79) 10 **12**	10 84% (0.63) 10 **12**	10 84% (0.66) 10 **13**
CONE (subsample)	10 92% (0.51) 10 **12**	10 96% (0.20) 10 **11**	10 94% (0.24) 9 **10**

Island model (scenario 1), hierarchical island model (scenario 2), and hierarchical stepping-stone model (scenario 3). Mod, modularity; CE, cross-entropy.

a We have reported the median, percentage of the number of correctly inferred subpopulations, an error measure (RMSE), and minimum and maximum (in boldface) values of the number of inferred subpopulations. We used both the modularity approach and StARS to choose a proper value for the tuning parameter *λ*. We have also reported the number of inferred clusters identified with the StARS subsampling (subsample).

**Figure 3:**
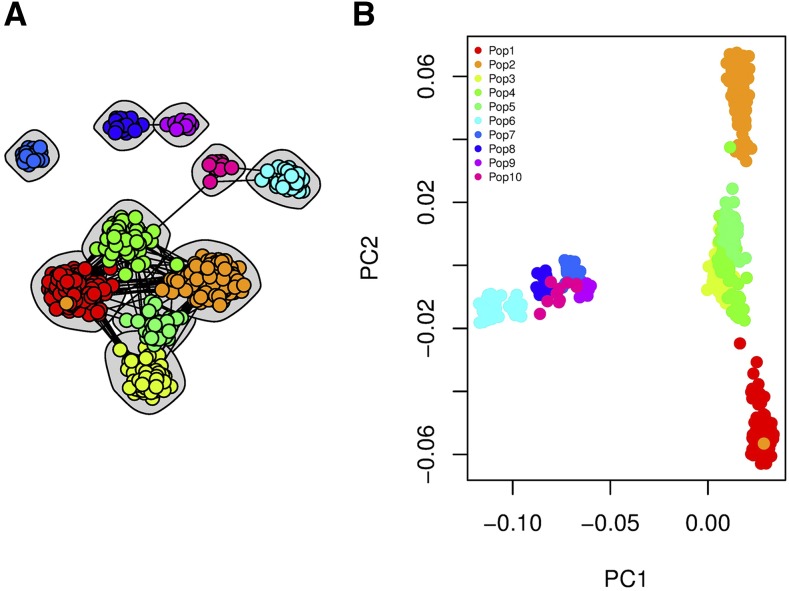
The population structure in hierarchical island model. (A) Detecting community structure through the unweighted graph estimate with no edge weights and the Walktrap algorithm. The tuning parameter was chosen via the modularity approach. (B) The PC1–PC2 plot of the same data set.

The essential part of our method is to inspect visually how different samples are connected at an individual level. Although CONE did not detect the upper hierarchical level clusters, in Figure 3A one can see how populations in the hierarchical island model from different archipelagos have more edges between them and are thus drawn more closely to each other. The community division graphically illustrated in Figure 3A is neatly in line with the PC1–PC2 plot presented in Figure 3B.

In most realistic settings there may be no well-defined notion of the “number of populations,” and in these settings the results are usually explored with different values of *K*. For example, both Evanno *et al.* (2005) and Puechmaille (2016) have shown that the STRUCTURE program usually detects the uppermost hierarchical structure in a sample. This hierarchy is not revealed with a distinct value of *K*. Therefore, inferring networks constructed with CONE by visual inspection is the easiest way to gain insight over the possible hierarchical structure of the data.

### Estimating the ancestry coefficients

To illustrate how CONE is able to estimate admixture coefficients compared to model-based clustering, we conducted a simulation study following the guidelines of Alexander *et al.* (2009) and Frichot *et al.* (2014). It should be noted that a fair comparison between the proposed method and model-based methods is not straightforward. Model-based methods describe the population structure by probabilistic assignment and our method characterizes the population structure with network communities. Nevertheless, paralleling our method with a state-of-the-art methodology serves as a good benchmark for our method’s potential. We compared our method to the model-based method sNMF.

**Figure 4:**
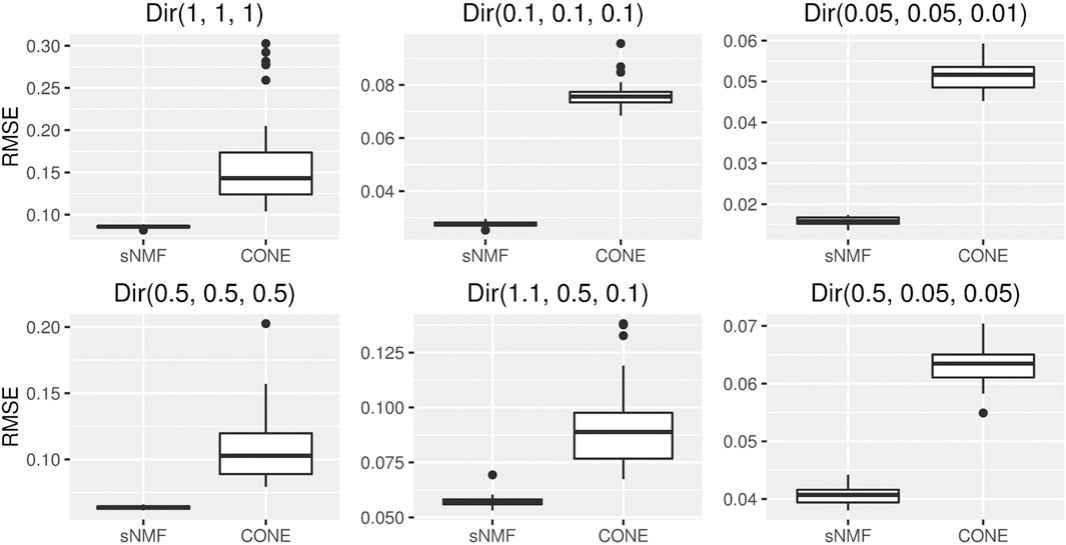
Accuracy of CONE and sNMF. Box plots of RMSEs between the estimated *Q* matrices and known ancestry proportions over 50 simulation runs with both sNMF and CONE.

With CONE, we used one data set as a validation set to choose a proper tuning parameter value with the modularity approach. Nevertheless, using the same value for the tuning parameter in a simulation run does not absolutely rule out the possibility that the dimensions of the true *Q* and the one estimated with the CONE framework differ. If CONE detected more (or less) than three populations, we modified the cut height of the dendrogram leading to three clusters. We used the RMSE to measure the accuracy of the estimated *Q* matrix, $\text{RMSE}\left(Q,\hat{Q}\right)={\left(NL\right)}^{-1/2}\times {\left\|Q-\hat{Q}\right\|}_{F},$ where ${\left\|A\right\|}_{F}$ is the Frobenius norm of an N×L matrix *A*. With sNMF, *K* was set to the true number of populations (three). We ran sNMF just once per each simulated data set. With sNMF, the value of the nonnegative regularization parameter *α* was set to either 0 or 100 according to the value that produced the smallest median value of the estimated RMSEs. The simulation results are presented in Figure 4.

**Figure 5:**
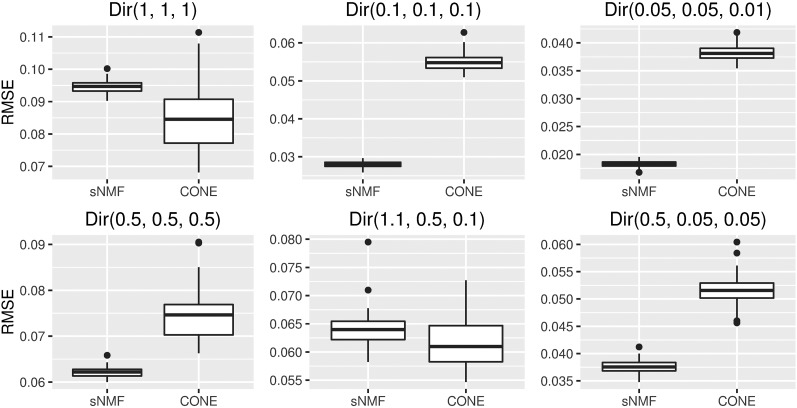
Accuracy of CONE (solution path) and sNMF. Box plots of RMSEs between the estimated *Q* matrices and known ancestry proportions over 50 simulation runs with both sNMF and CONE.

**Figure 6:**
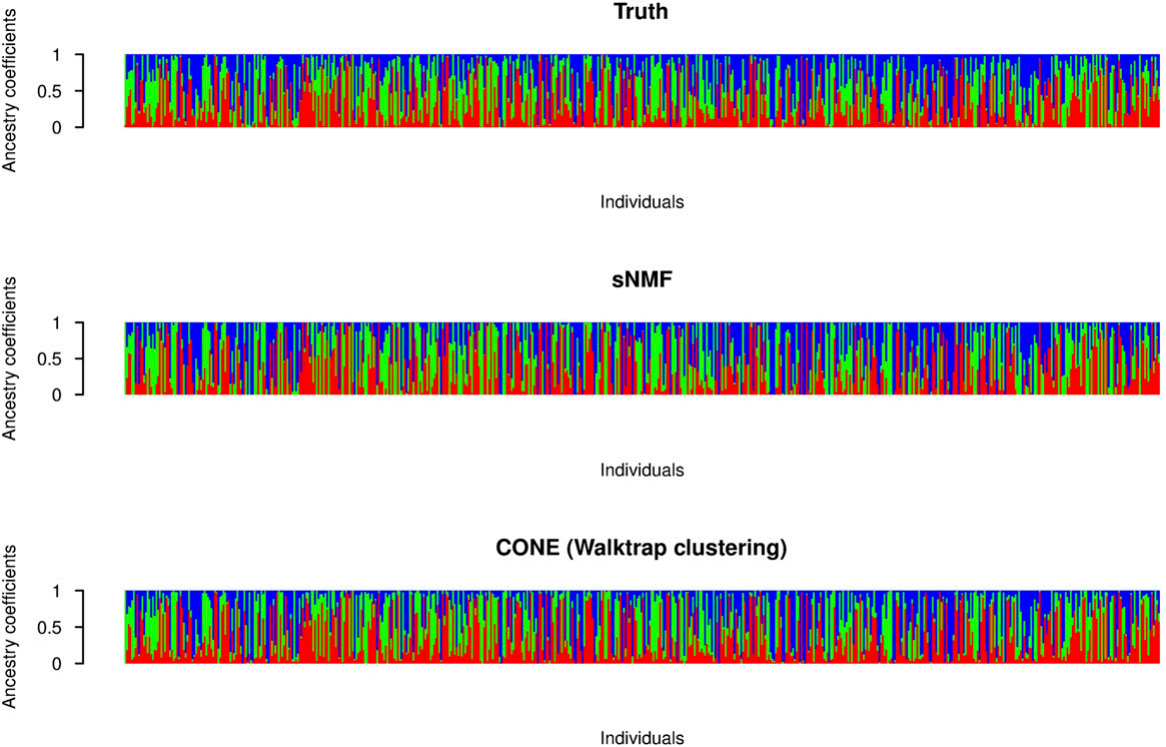
Graphical representation of ancestry coefficients. Simulated ancestry coefficients and the corresponding estimates computed with sNMF and CONE from an arbitrary simulated data set. The entries of the *Q* matrix considered here were generated from the Dir(0.5,0.5,0.5) distribution.

**Figure 7:**
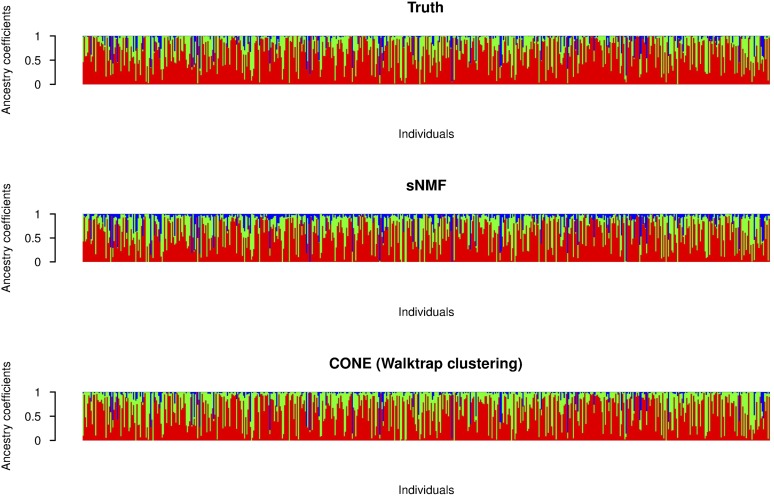
Graphical representation of ancestry coefficients. Simulated ancestry coefficients and the corresponding estimates computed with sNMF and CONE from an arbitrary simulated data set. The entries of the *Q* matrix considered here were generated from the Dir(1.1,0.5,0.1) distribution.

**Figure 8:**
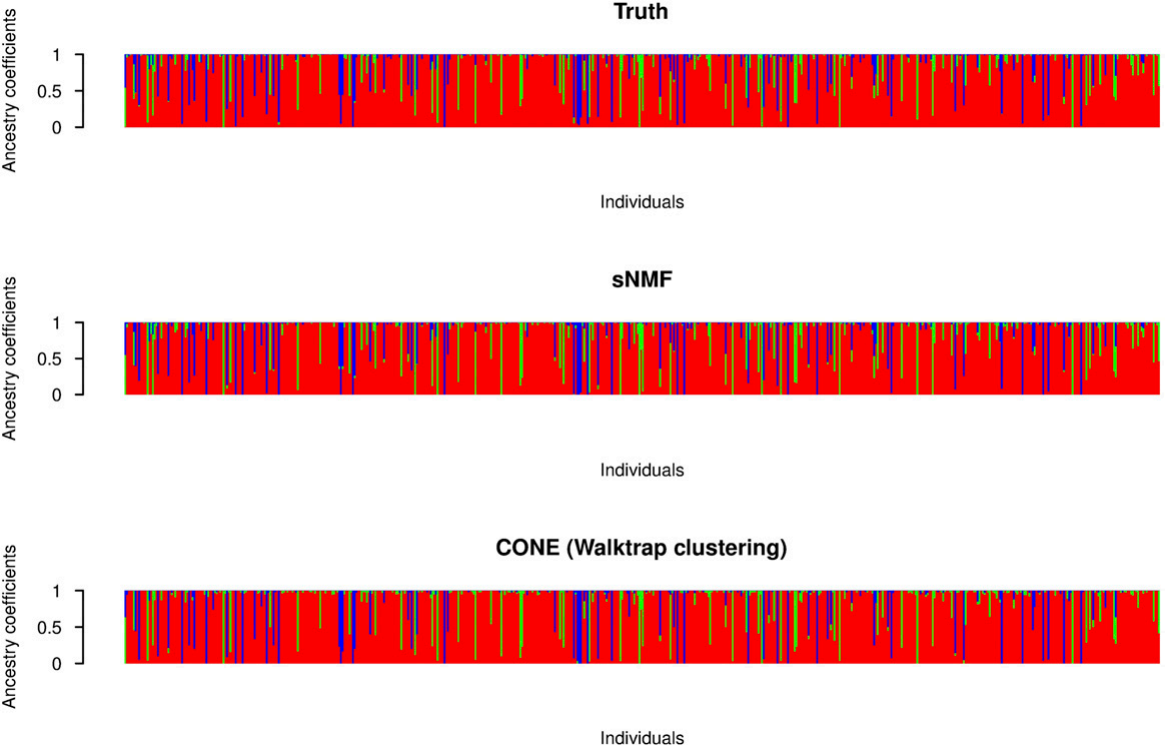
Graphical representation of ancestry coefficients. Simulated ancestry coefficients and the corresponding estimates computed with sNMF and CONE from an arbitrary simulated data set. The entries of the *Q* matrix considered here were generated from the Dir(0.5,0.05,0.05) distribution.

Using sNMF, accurate ancestry estimates with completely admixed and unrelated individuals were obtained for all simulated population models. The error measures estimated with sNMF are in line with previous studies (Alexander *et al.* 2009; Frichot *et al.* 2014; Raj *et al.* 2014). A larger dispersion of the CONE results can be explained by the fact that we did not optimize the tuning parameter in each simulated data set separately. To give a better illustration of CONE’s potential, we performed an additional analysis by determining the ancestry estimates with several values of the tuning parameter *λ*. Then, we choose the estimated $Q_{\lambda }$ which minimized the RMSE of the ancestry estimate. The results are presented in Figure 5. In each case, error estimates of CONE were diminished in every parameterization of the Dirichlet distribution. CONE even seemed to be able to give more accurate ancestry proportion estimates than sNMF in two examined parameterizations of the Dirichlet distribution. We have graphically represented typical results of the ancestry coefficients estimated with both sNMF and CONE in Figure 6, Figure 7, and Figure 8.

We find it remarkable that CONE was able to estimate ancestry coefficients even close to those obtained by model-based methods, such as sNMF. With CONE, we did not make any assumptions over the relationship of the *Q* matrix and the binomial model used in our simulation analysis. The relatively small error estimates suggests that a sparse individual-level network represents different levels of admixture in the data.

### Application to teosinte data

To illustrate how CONE is able to describe population structure in a practical application, we applied it to moderately sized SNP genotype data sets previously described in Pyhäjärvi *et al.* (2013).

**Figure 9:**
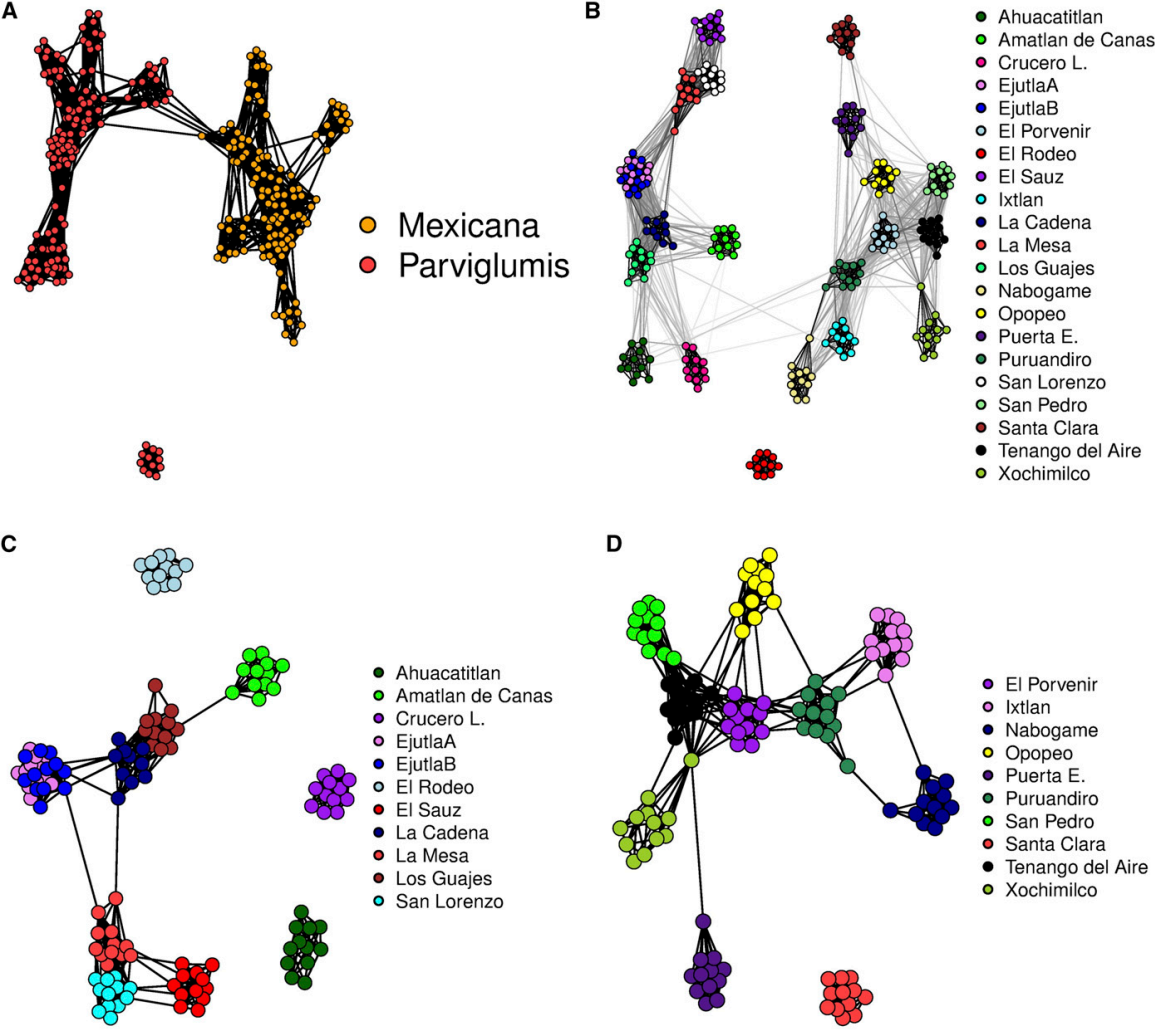
The individual-level dependency structure between different samples in the teosinte data. (A) Graph constructed by using our customized neighborhood selection scheme and the nodes are colored according to the subspecies they represent. (B) Weighted graph in which the additional StARS weights are used as edge weights; the nodes are colored based on the 21 populations identified in the teosinte data. (C) Network of the *parviglumis* subspecies. (D) Network of the *mexicana* subspecies. The coloring and population labels were added after graph estimation in all cases.

We started our analysis of these data sets by running the StARS procedure with 20 subsamples each containing almost 2000 SNP markers, performing the logistic multinomial LASSO regression on each subsample in turn. 40 different values of the tuning parameter *λ* ranging from 0.05 to 0.5 were tested. Using our in-house R code, it took ∼30 min to perform the StARS procedure for both of the subspecies data sets on a standard desktop computer, and ∼90 min for the complete data set. After completing the StARS analysis, we used all of the SNP markers in the neighborhood selection procedure. This graph construction process took just a few minutes, and was finalized using the Fruchterman–Reingold algorithm as implemented in the qgraph package to delineate different communities. The final graphs, which we refer to as individual-level *dependency networks*, are presented in Figure 9.

Figure 9, A and B, clearly shows that the samples are divided into two major subpopulations according to the two subspecies and one minor subpopulation (the El Rodeo population). The network shown in Figure 9B clearly indicates that, while the samples are divided into several populations, these populations are arranged into two larger groups corresponding to the two subspecies, and gene flow occurs (or has occurred) mostly within these groups. Overall, the Walktrap algorithm divided the samples into 12 different communities. On the other hand, the network modularity revealed that the optimal number of different communities would be ∼12–20 in the data set (Figure S1). In the STRUCTURE analysis from Pyhäjärvi *et al.* (2013), where the number of populations was set to four, the El Rodeo subpopulation from *parviglumis* stands out as a separate population (their figure 4B). The divergence of the El Rodeo subpopulation is probably due some environmental factor; the edge lengths in our dependency network illustrate only the strength of the genetic relationship between individuals, not the physical distance. For example, the Nabogame population in both Figure 9, B and D, is connected to other populations from the same subspecies, even though the corresponding samples are widely separated from other *mexicana* populations (figure 1 of Pyhäjärvi *et al.* 2013). The graphs in Figure 9, A and B, indicate that there is some gene flow between the subspecies and that most of it occurs between the Ahuacatitlan and Puruandiro populations: this is consistent with the STRUCTURE analysis presented by Pyhäjärvi *et al.* (2013) (their figure 4B). As in their analysis, the Ahuacatitlan population remains strongly connected to other *parviglumis* populations, but there are only a few weak edges connecting it to *mexicana* populations. Our graph interpretation therefore does not support the hypothesis that Ahuacatitlan is a sister group to *mexicana*. In Figure 9, B and C, it can be seen that the two Ejutla populations merge into a single population. This result is also consistent with the PCA of Pyhäjärvi *et al.* (2013) and, on the whole, our depencency network shown in Figure 9B has the same characteristics as the PC1–PC2 plot of Pyhäjärvi *et al.* (2013) (their figure 4A). As mentioned above, the *mexicana* populations include two hybrid samples. We assume that hybrid samples are the two nodes from Puruandiro and Nabogame which connect these two populations in Figure 9D. Estimated ancestry coefficients with K=12 and K=17 are graphically represented in Figure S2.

### Application to E. coli data

As a second practical example we evaluated the performance of our method by applying it to the SNP genotype data set previously described in Salipante *et al.* (2015).

**Figure 10:**
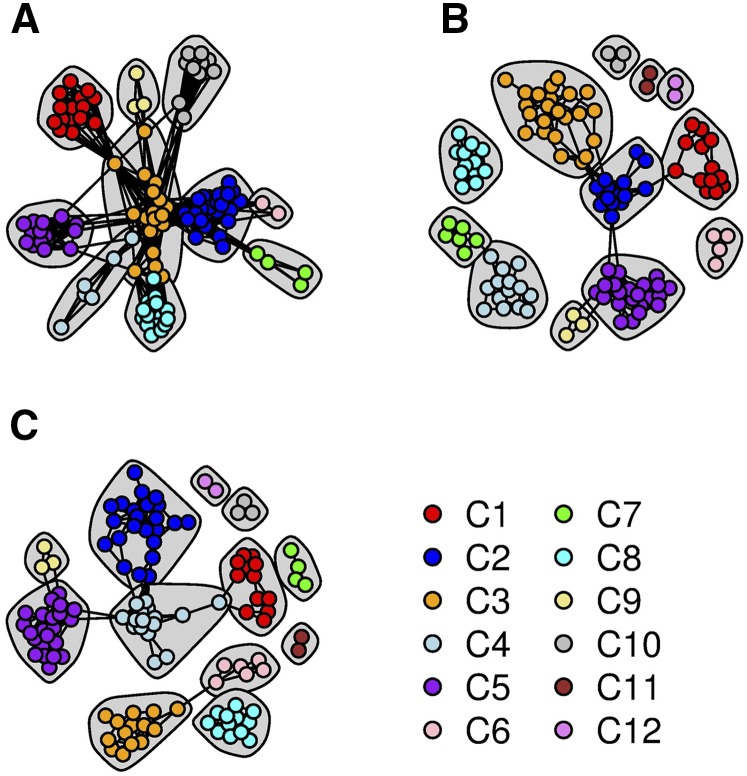
The dependency structure inferred between individuals from the *E. coli* data with the CONE framework. Each strain is represented by a colored node and labeled based on the division of the samples into 12 different communities (C1, C2, …, C12) detected with CONE. (A) Additional graph estimated using the StARS procedure. (B) The graph resulting from CONE framework. (C) The weighted graph obtained by combining the two aforementioned graphs.

We began the CONE procedure with the *E. coli* data set by running the StARS procedure with 20 subsamples containing 5775 SNP markers each, performing the logistic multinomial LASSO regression to each subsample individually. We used 40 different values for the tuning parameter *λ* ranging from 0.05 to 0.5, and it took ∼90 min to complete the StARS procedure using our in-house R code. After the StARS analysis, we used all the SNP markers in the neighborhood selection procedure. Running the neighborhood construction process took ∼208 min to complete. As in the teosinte graph construction, we used the Fruchterman–Reingold algorithm, as implemented in the qgraph package, to distinguish different communities from each other for visual representation. The final graph estimates are presented in Figure 10.

**Figure 11:**
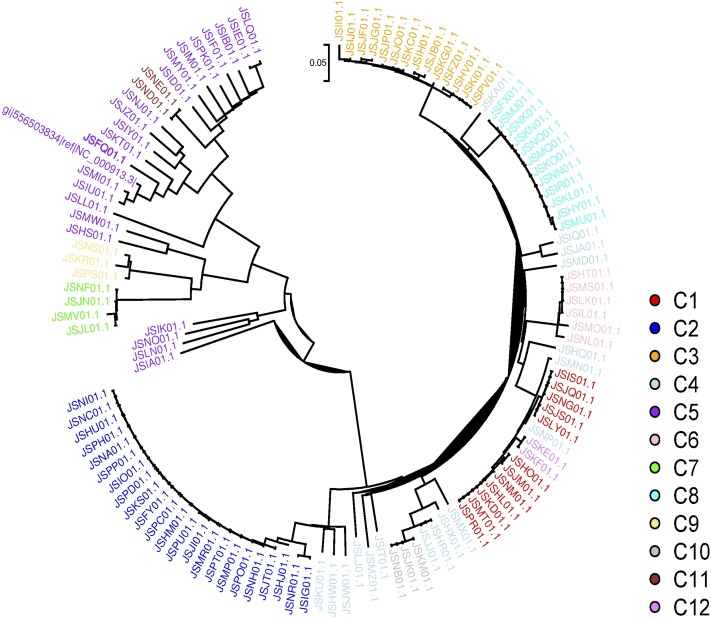
The phylogenetic tree generated from the *E. coli* data. Strains are colored according to the community division presented in Figure 10.

In Figure 10, we colored the nodes according to their community membership. Community detection was done using the Walktrap algorithm. Based on the graph estimates, we concluded that there are 10–12 different communities represented in the complete data set. We did not include the strain IDs in the networks shown in Figure 10, but a network including the strain IDs is provided in Figure S3 for readers seeking more detailed information on the nodes. After making the division, we used the same coloring scheme to color the phylogenetic tree that we derived from the data; this phylogenetic tree is presented in Figure 11, and a more detailed version can be seen in Figure S4.

**Figure 12:**
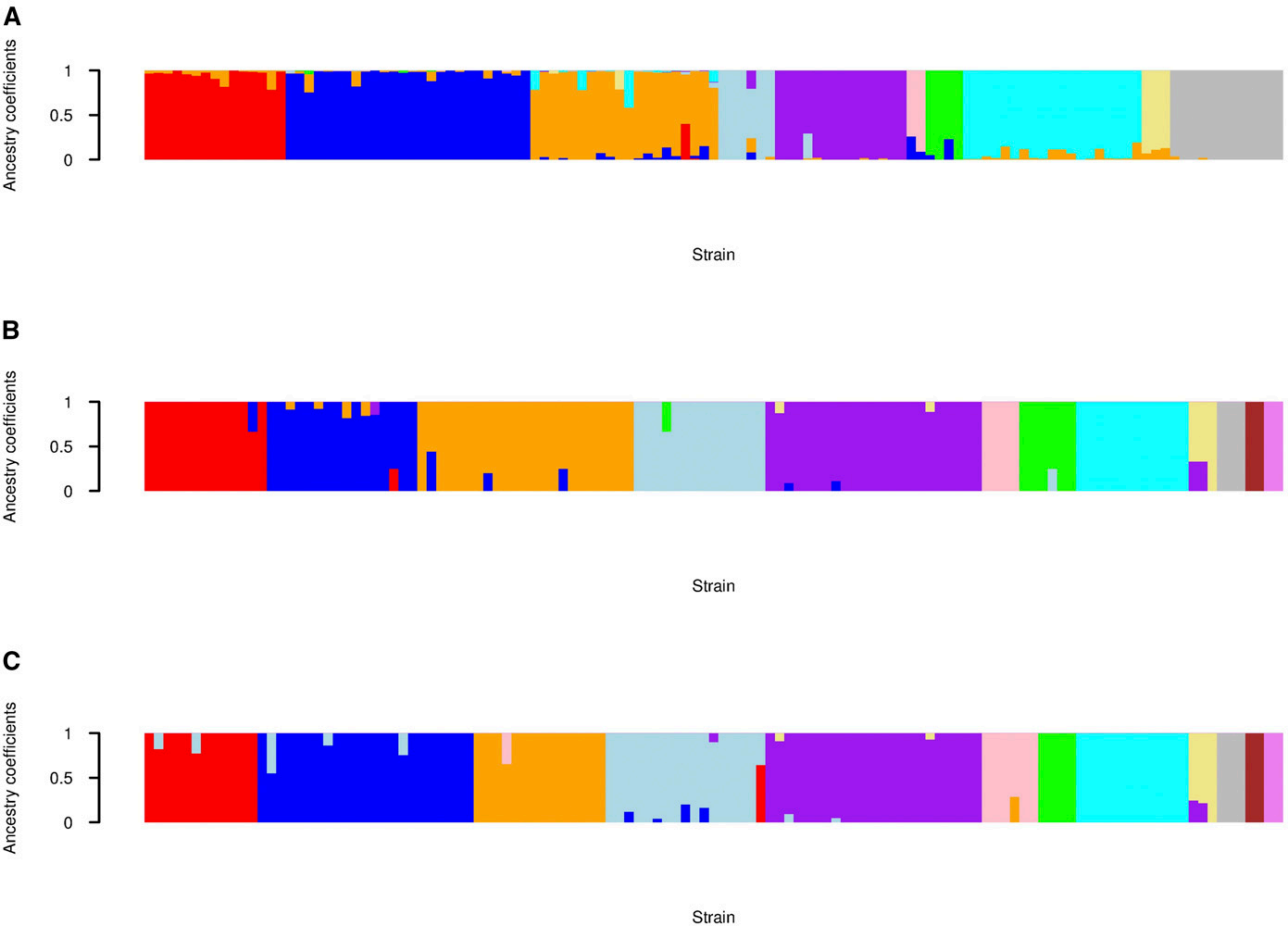
Graphical representation of the estimated ancestry coefficients obtained from the *E. coli* data set. (A) The estimated ancestry coefficients using the weighted network (Figure 11A). With this network estimate, the number of detected clusters was 10 ($\hat{K}$ = 10). (B) The estimated ancestry coefficients using the unweighted network (Figure 11B). (C) The estimated coefficients using the weighted network (Figure 11C). With network estimates (B and C), the number of detected clusters was 12 ($\hat{K}$ = 12). Different clusters are colored according to the same coloring presented in Figure 10 and Figure 11.

As clearly shown by Figure 10 (see also Figure S3 for the strain names), the inferred community partition resembles the clades in the phylogenetic tree (Figure 11). The resemblance is especially apparent when comparing the phylogenetic tree to communities C2, C3, C7, and C8. Moreover, the nodes linking communities C2 and C4 in Figure 10, A–C, are reflected in the branch connecting strains from the corresponding communities in Figure 11. However, there are some minor differences between the tree’s branch structure and the node arrangement in Figure 10, B and C, which are based on analyses of the complete data set. For example, community C8 appears to be isolated, with no edges linking it to C3. However, in the phylogenetic tree, there are some branches connecting strains from these communities, while their genetic relationship is weak. In the StARS graph, there are some wavering edges between communities C3 and C8 (Figure 10A). Additionally, the small communities identified by the CONE analysis (C7, C9, C10, C11, and C12) are separated from the other communities; this division is somewhat consistent with Figure 11 because strains from communities C7 and C11 are only distantly related to other strains. In addition, we computed the ancestry coefficients with CONE (Figure 12).

### Application to HGDP data

To emphasize the potential of the CONE framework in the analysis of very large SNP data sets, we applied it to the so-called Stanford HGDP SNP genotyping data set (Rosenberg *et al.* 2002), which has previously been analyzed by authors including Li *et al.* (2008) and Raj *et al.* (2014) using FRAPPE, ADMIXTURE, fastSTRUCTURE, and PCA. Overall there are 1043 individuals from 51 different populations and 660,918 SNP markers.

**Figure 13:**
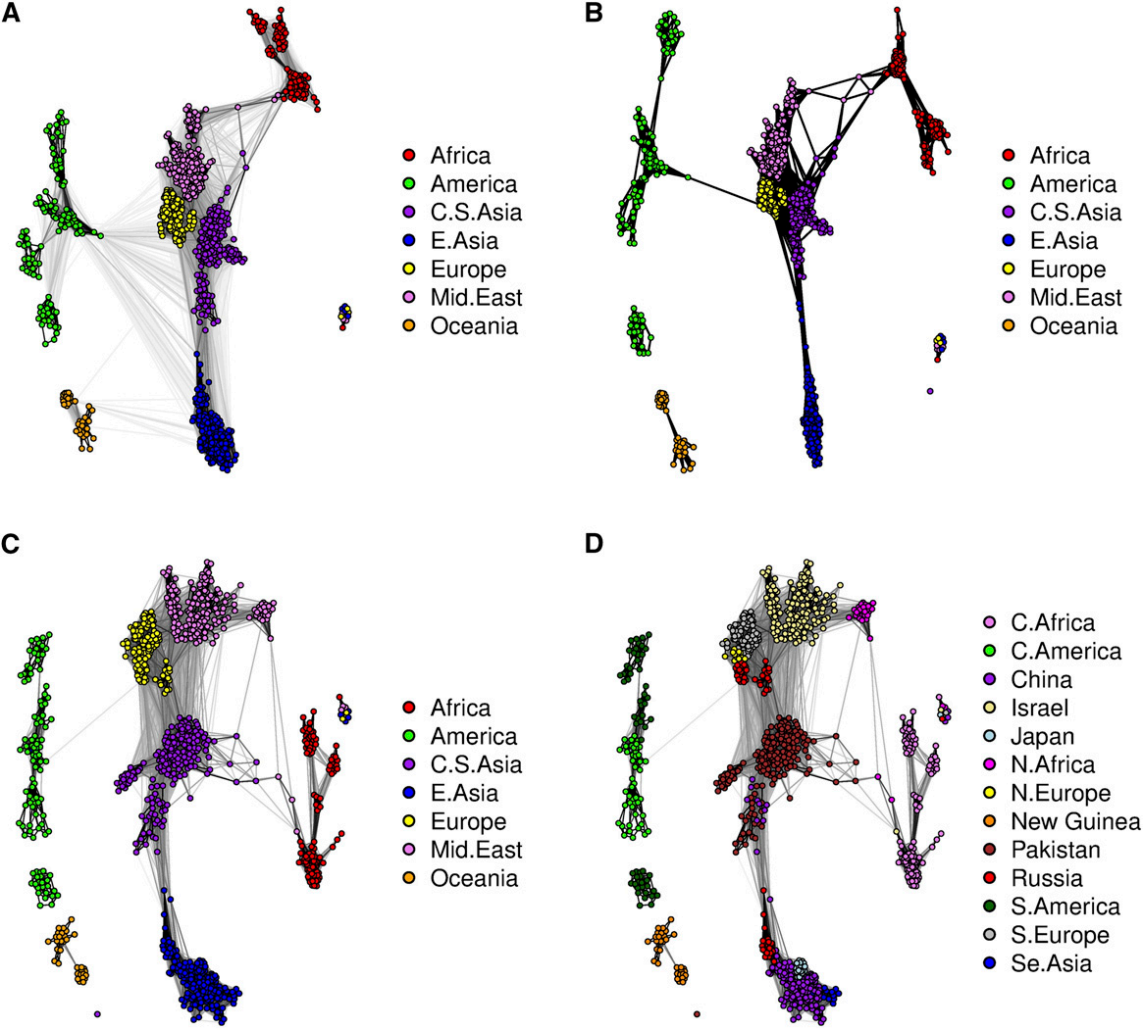
The dependency structure between individuals in the HGDP data set inferred with CONE framework. (A) The additional graph estimated from the StARS procedure. (B) The customized neighborhood selection graph. (C) The weighted graph obtained by combining the two. (D) Graph colored according to the different geographic areas from which the individuals in the data set originate. Each individual is represented by a colored node according to the division of the data into seven different populations as described by Raj *et al.* (2014). The associated coloring and population labels were added after the graph was estimated.

As with the teosinte data, the first step in the CONE procedure was to perform the StARS procedure with 20 subsamples, each containing 8120 SNP markers, applying the logistic multinomial LASSO regression to each subsample separately. We used 40 different values for the tuning parameter *λ*, ranging from 0.05 to 0.5. It took ∼2 d to run StARS analysis in R. We ultimately set the value of the tuning parameter $\hat{\lambda }$ to 0.0812. Using this value we obtained our first graph estimate, which is presented in Figure 13A.

After the StARS run, we analyzed the full data set (all 659,421 SNPs) using the optimized $\hat{\lambda }$ value. Having obtained the corresponding adjacency matrix, we used the Fruchterman–Reingold algorithm to detect different communities among the nodes, yielding the graph estimate presented in Figure 13B. As was done with the teosinte data, we used this graph along with the additional StARS graph and calculated the entrywise product of the corresponding adjacency matrices to produce an emphasized graph estimate: These graph estimates are presented in Figure 13, B and C. To better illustrate the details of the population group division, we colored the graph in Figure 13D according to the geographic areas associated with the samples. Again, we will refer to the graphs presented in Figure 13, A–D, as dependency networks. It took almost 4 d to perform our customized neighborhood selection procedure on the whole data set using glmnet with R running on a desktop computer with 8 GB of RAM and a 3.1 GHz Intel Core i5-2400 processor. In our opinion, this demonstrates the CONE framework’s ability to analyze large data sets in reasonable time frames even when using only moderately powerful hardware and readily available software.

The estimated networks have similarities with the ancestry proportions estimated by fastSTRUCTURE and ADMIXTURE, as reported by Raj *et al.* (2014), and with the PCA analysis presented by Li *et al.* (2008). The graphs in Figure 13 show that the Middle Eastern populations are closely related to the European populations. There are also clear indications of gene flow between the populations living in Eurasia and North Africa, which is consistent with the PC1–PC2 plot (their figure S3B) presented in the supplemental material of Li *et al.* (2008). Both the Oceanian and American populations are estimated to belong to separate groups despite the presence of minor gene flow between Europe and America, as indicated by both the ADMIXTURE and fastSTRUCTURE analyses. The fastSTRUCTURE and ADMIXTURE estimates differ from each other with respect to the way they divide the African and Native American populations. The dependency networks also indicate that both the American and African populations are divided into multiple subgroups. The ADMIXTURE ancestry proportions imply the existence of two distinct American population groups with some gene flow between the Northern and Southern populations; this is consistent with our graph estimates, in which there are some edges linking Southern and Central American individuals. Moreover, the fastSTRUCTURE ancestry proportions indicate that the African populations are split into Northern and Central African groups, with some gene flow between them. Both the ADMIXTURE analysis and the dependency networks imply the existence of a weak gene flow between the Central Asian and African populations. The PCA analysis of Li *et al.* (2008) suggests that the Oceanian populations are divided into two subpopulations, which is again consistent with the dependency networks. Neither the ADMIXTURE nor the fastSTRUCTURE analyses revealed a similar division.

**Figure 14:**
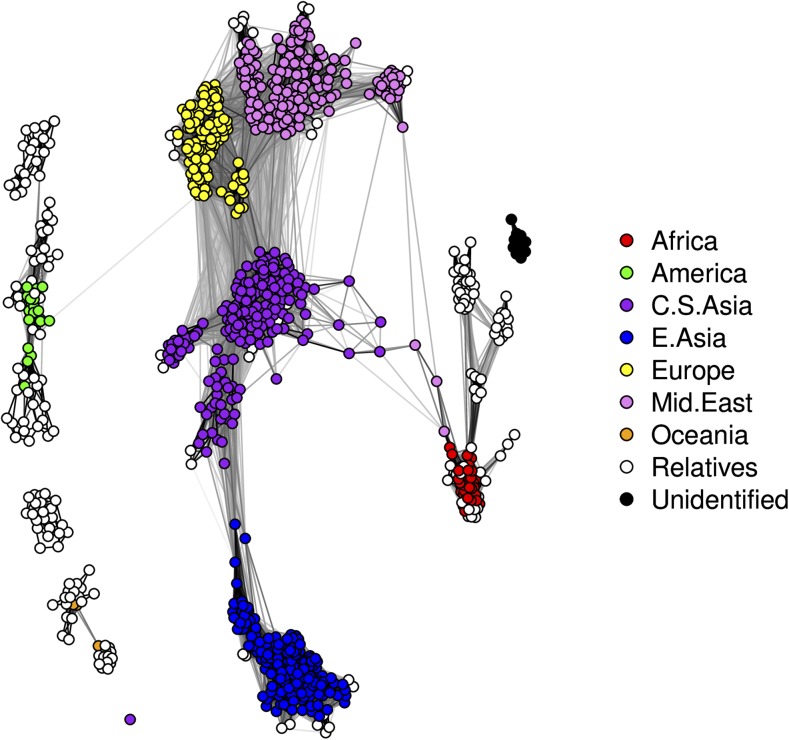
The dependency structure between individuals in the HGDP data set as inferred with CONE. Closely related individuals (white nodes) were identified by considering the genomic relationship matrix.

To determine how the inclusion of close relatives affected our graph estimation, we computed the genomic relationship matrix (VanRaden 2008). In Figure 14, nodes corresponding to closely related individuals are shown in white. As can be seen, close relatives are present in every population group but their presence did not seem to adversely affect the neighborhood selection process.

**Figure 15:**
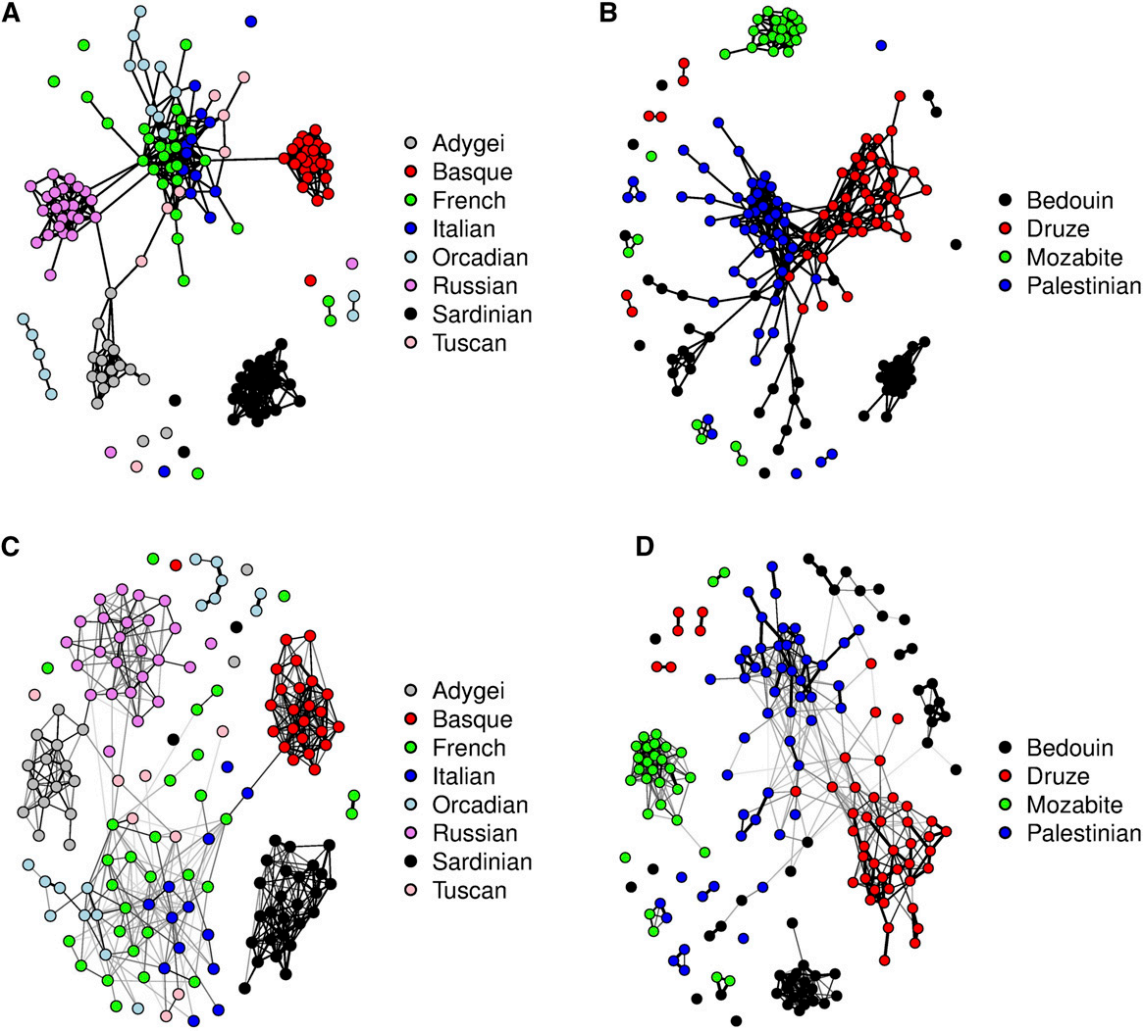
The dependency structure inferred with CONE of a subsample of the HGDP data. (A) Unweighted graph of the European populations. (B) Unweighted graph of the Middle East populations. (C) Weighted graph of the European populations. (D) Weighted graph of the Middle East populations.

To further compare our results to those of Li *et al.* (2008), we also performed a fine-scale population graph analysis of the European and Middle Eastern populations. Figure 15 shows the results obtained by analyzing the European and the Middle Eastern populations with CONE, which can be compared to the PCA analysis of Li *et al.* (2008). The graphs in Figure 15, C and D, are so-called emphasized graphs, which were constructed in a similar way to those shown in Figure 13, C and D, and Figure 14. Both graphs are quite similar to the PC1–PC2 plots of Li *et al.* (2008) (figure 2, A and B, in their manuscript). As noted in Li *et al.*’s discussion of the PCA results, the European populations can be separated into four relatively separate subpopulations (Adygei, Basque, Russian, and Sardinian), and a number of seemingly independent individuals are present. In Figure 15B, the Bedouins seem to divide evenly into three continuous groups, with some separated individuals indicating weak gene flow between other populations. The Mozabite are distinguished as a separate population, which is consistent with the structure of the North African region in the emphasized graph shown in Figure 15D.

There are some elements of the dependency networks that are not intuitively clear and whose interpretation would require a detailed analysis of the data. The first oddity is that one Middle Eastern individual was appointed to the central African population with no direct connection to any other individual among the Middle Eastern populations. Another difficult-to-interpret subpopulation is the small population group consisting of 12 samples from various backgrounds that is apparent in Figure 13 and Figure 14. The elements of the genomic relationships between these individuals are also quite large (>0.71), so these samples were at least partly excluded in previous analyses of the data set (Li *et al.* 2008; Raj *et al.* 2014). We have listed these individuals in Table 3 and indicated them using black nodes in Figure 14. We suggest that the formation of this extra group is not due to problems with the CONE procedure and instead indicates that these individuals have interesting qualities that have not previously been identified. Only one individual (from the Pathan population, ID no. 00251 in the HGDP data set) was estimated to be totally independent from all of the other populations.

**Table 3:** Twelve outlier individuals that differ remarkably from other individuals from the corresponding populations

HGDP ID	Population	Geographic Area
01275	Mozabite	North Africa
00904	Mandenka	Central Africa
01269	Mozabite	North Africa
00685	Palestinian	Israel
00737	Palestinian	Israel
01223	Mongolia	China
01078	Sardinia	Southern Europe
00750	Japanese	Japan
01023	Han	China
01076	Sardinia	Southern Europe
01322	Lahu	China
00889	Russia	Russia

As also found by Raj *et al.* (2014), both fastSTRUCTURE and ADMIXTURE analyses suggest that more than seven population groups are represented in the data. The dependency networks also indicate that there may be more than seven population groups. Estimated ancestry coefficients with different values of *K* (K=20, K=21, and K=25) are graphically represented in Figure S5 and Figure S6.

## Discussion

By applying the CONE method to certain simulated scenarios of population history as well as real-world data sets previously analyzed using PCA (Price *et al.* 2006), STRUCTURE (Pritchard *et al.* 2000; Falush *et al.* 2003), ADMIXTURE (Alexander *et al.* 2009), fastSTRUCTURE (Raj *et al.* 2014), and FRAPPE (Tang *et al.* 2005), we have demonstrated that graph inference using neighborhood selection can be used for individual-level population structure analysis based on SNP data.

When applied to the *E. coli* data set, our network estimation method gave results that were consistent with the phylogenetic tree. In general, the inferred community partition closely resembled the distribution of clades within the tree. The potential for recombination among and within lineages in the *E. coli* population could give rise to many difficulties in estimating its relatedness structure, which is a well-known limitation in phylogenetic tree reconstruction (Posada and Crandall 2002). Population graphs constructed using our method could help to resolve such problems because the observation of multiple connections between strains coupled with the graph’s topology can reveal deviations from the clonal frame (corresponding to the absence of recombination).

It has been shown that MB-style approximation is also consistent in the so-called high-dimensional setting as the number of continuous Gaussian random variables increases (Meinshausen and Bühlmann 2006). When using our graphical approach, the high-dimensional scenario would correspond to an uncommon case in which there are more individuals than measured “genotypes” in the data set. We did not examine the analytic properties of such settings, but they could potentially cause some practical problems in the StARS procedure, because the subsampling in StARS can give rise to very uneven allele frequencies; leading to failures of convergence when attempting to obtain maximum likelihood estimates. It may also be the case that the number of falsely estimated neighbors would make meaningful interpretation of the network impossible. We also examined the potential for graphical structure analysis based on multiallelic microsatellite data but found the CONE procedure to be unusable with such data sets that include large and variable numbers of classes.

Our method is able to provide a *Q* matrix (*i.e.*, population membership probabilities for the individuals in the data), which could subsequently be used in an association analysis model to correct for population stratification (Yu *et al.* 2006). However, it is completely unknown to us how well it may work in practice and therefore we emphasize that one should proceed with caution. Further examination of the relationship between the ancestry coefficients and network estimation should be derived for more exact results, but further dwelling to the subject is left for future studies.

Jalali *et al.* (2011) have shown that for a multinomial model, one can use a group LASSO penalty whereby the regression coefficients corresponding to the same explanatory variable (individual) for all three classes are forced to all become zero or nonzero together. Using the group LASSO penalty would also yield a consistent neighborhood selection procedure for the edge set ε under strict conditions. However, Jalali *et al.* (2011) used cross-validation to choose the tuning parameter *λ*. In our experience, cross-validation tends to produce overly dense graphs that are hard to interpret. The glmnet package also includes the option to apply group LASSO penalties to the coefficients. We tested this penalization scheme in an analysis of the European and Middle Eastern populations from the HGDP data, using the StARS procedure to choose an optimal value for the regularization parameter *λ*. In the case of the European populations, the results were practically identical to those obtained using our simpler conditions for neighborhood selection (Figure S7). For the Middle Eastern populations, the biggest difference was that the group LASSO penalty seemed to estimate individuals belonging to the Druze and Palestinian populations to be more closely related than was the case without penalization. The major drawback of the group LASSO approach is that it increases the time required to run the neighborhood selection procedure. For example, the StARS analysis for the Middle Eastern populations took ∼2.5 hr with our simple edge selection rule whereas the group LASSO method took >9 hr to complete. When dealing with large SNP panels, because the choice between the group LASSO penalty and our rule-based approach seems to have minimal influence on the individual-level population graphs, the faster computation time favors our rule-based approach over the group penalty. Moreover, it is important to note that the dependency networks seem to be much more sensitive to the way of choosing the tuning parameter (StARS in our case) than to the use of a group penalty.

We are interested in developing our method further and reducing its computational time. Novel statistical methods such as BIGQUIC (Hsieh *et al.* 2013) have been proposed to handle large data sets rapidly. Similar to our approach, BIGQUIC can analyze data with $10^{6}$ variables with the R program. Parallel computing speeds BIGQUIC up even more. Implementing our method in user friendly software is left for future studies.

The methods described here have been implemented using R and our own procedures. A collection of codes can be found in File S1.

## Supplementary Material

Supplemental material is available online at www.g3journal.org/lookup/suppl/doi:10.1534/g3.117.300131/-/DC1.
