# Supplementary material for: CONE: Community Oriented Network Estimation Is a Versatile Framework for Inferring Population Structure in Large-Scale Sequencing Data
Source: G3 (Bethesda). 2017 Aug 22;7(10):3359–77. doi: 10.1534/g3.117.300131 (PMC5633386; doi:10.1534/g3.117.300131)
Supplement: Supplementary file 1 [file 3359FigureS1.pdf]

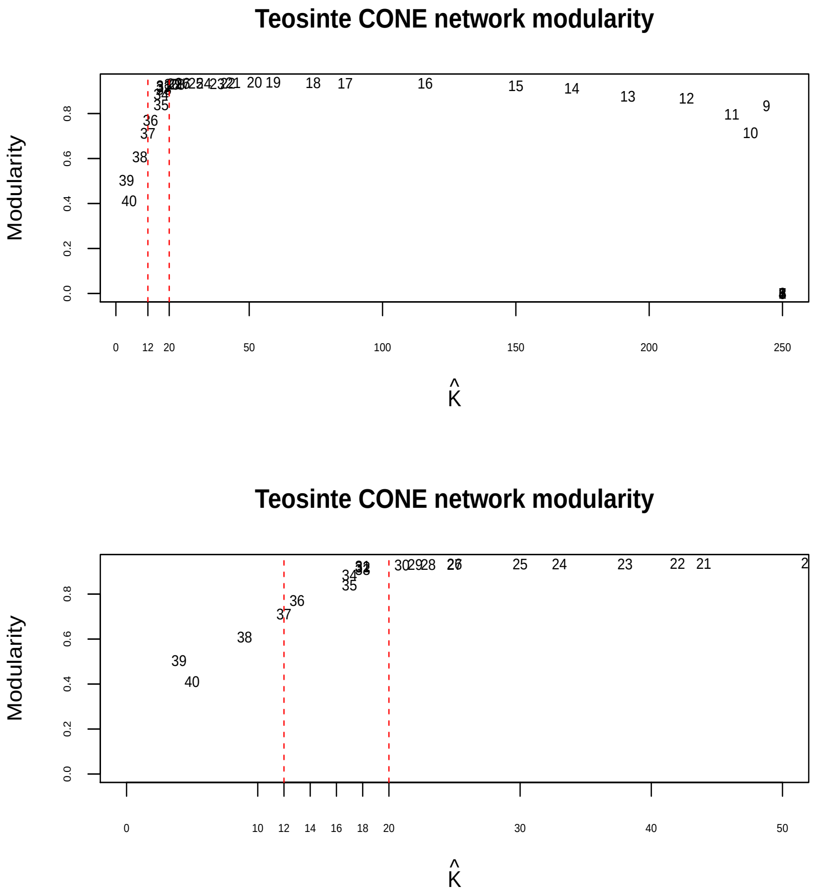

Network modularity plotted against the inferred number of clusters (communities) in the teosinte data. The chosen number of clusters is the number of clusters where the network modularity starts to decline (an “elbow”). The lower panel is the scaled version of the upper panel.
