# Supplementary material for: CONE: Community Oriented Network Estimation Is a Versatile Framework for Inferring Population Structure in Large-Scale Sequencing Data
Source: G3 (Bethesda). 2017 Aug 22;7(10):3359–77. doi: 10.1534/g3.117.300131 (PMC5633386; doi:10.1534/g3.117.300131)
Supplement: Supplementary file 2 [file 3359FigureS2.pdf]

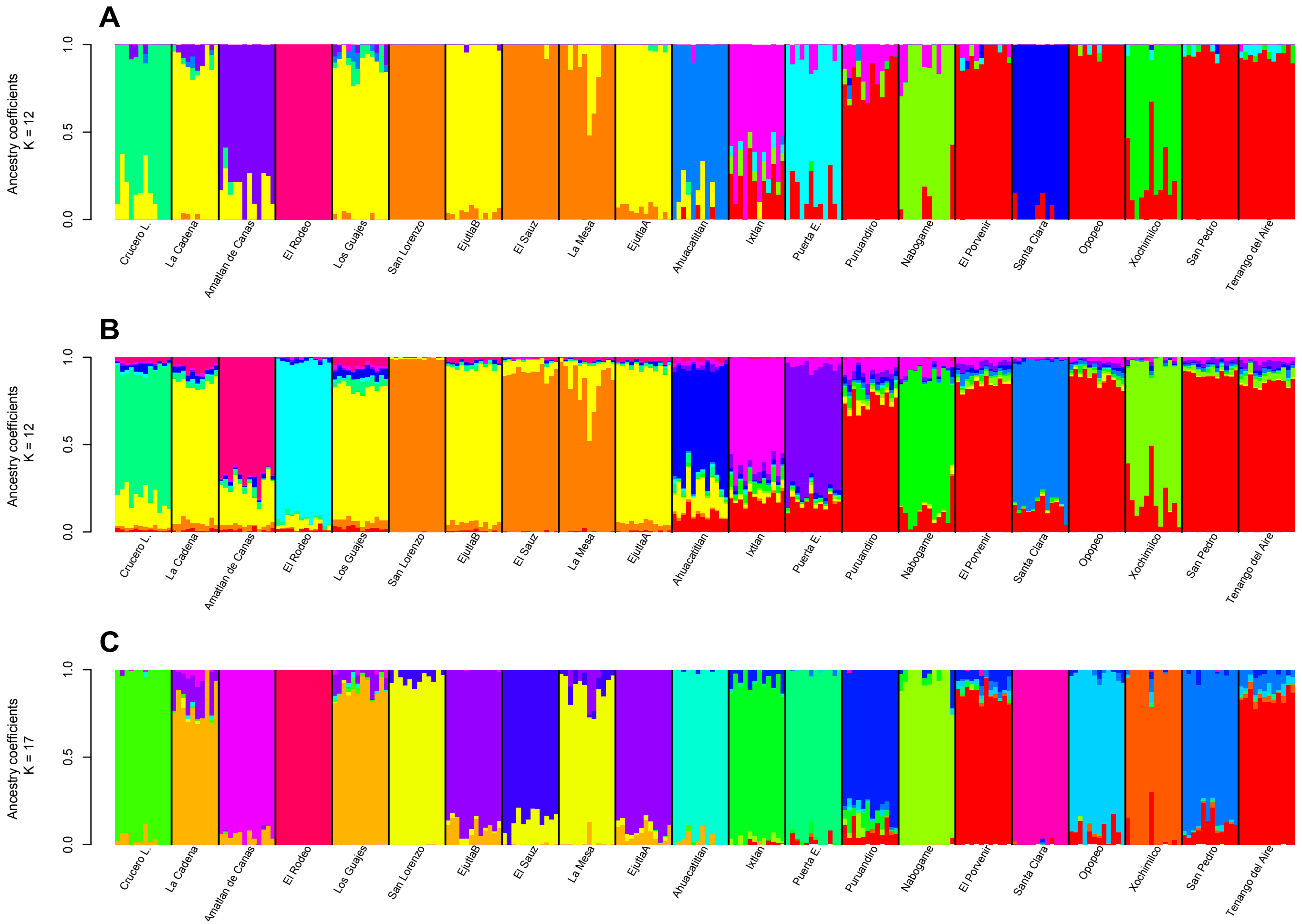

Ancestry coefficients inferred with CONE in the teosinte data. (**A**) Estimated ancestry coefficients using the community structure inferred in the unweighted network with $K = 12$. (**C**) Estimated ancestry coefficients using the community structure in the weighted network (StARS subsampling) with $K = 12$. (**C**) Estimated ancestry coefficients using the community structure inferred in the weighted network computed by combining both previously mentioned networks with $K = 17$.
