# Supplementary material for: CONE: Community Oriented Network Estimation Is a Versatile Framework for Inferring Population Structure in Large-Scale Sequencing Data
Source: G3 (Bethesda). 2017 Aug 22;7(10):3359–77. doi: 10.1534/g3.117.300131 (PMC5633386; doi:10.1534/g3.117.300131)
Supplement: Supplementary file 3 [file 3359FigureS3.pdf]

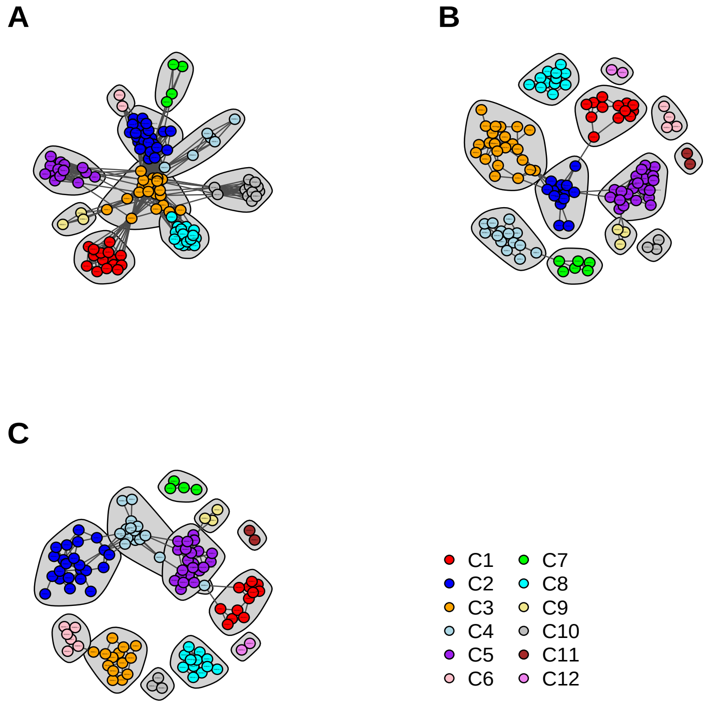

The dependency structure inferred between samples from the *E. coli*. Each strain is represented by a colored node and labeled based on the division of the samples into 12 different communities (C1, C2, . . . , C12) detected with CONE. Inferred network communities are illustrated with shaded areas. Each node is labeled with the corresponding strain ID. (A) Additional graph estimated using the StARS procedure. (B) The graph resulting from CONE framework. (C) The weighted graph obtained by combining the two aforementioned graphs.
