# Supplementary material for: CONE: Community Oriented Network Estimation Is a Versatile Framework for Inferring Population Structure in Large-Scale Sequencing Data
Source: G3 (Bethesda). 2017 Aug 22;7(10):3359–77. doi: 10.1534/g3.117.300131 (PMC5633386; doi:10.1534/g3.117.300131)
Supplement: Supplementary file 4 [file 3359FigureS4.pdf]

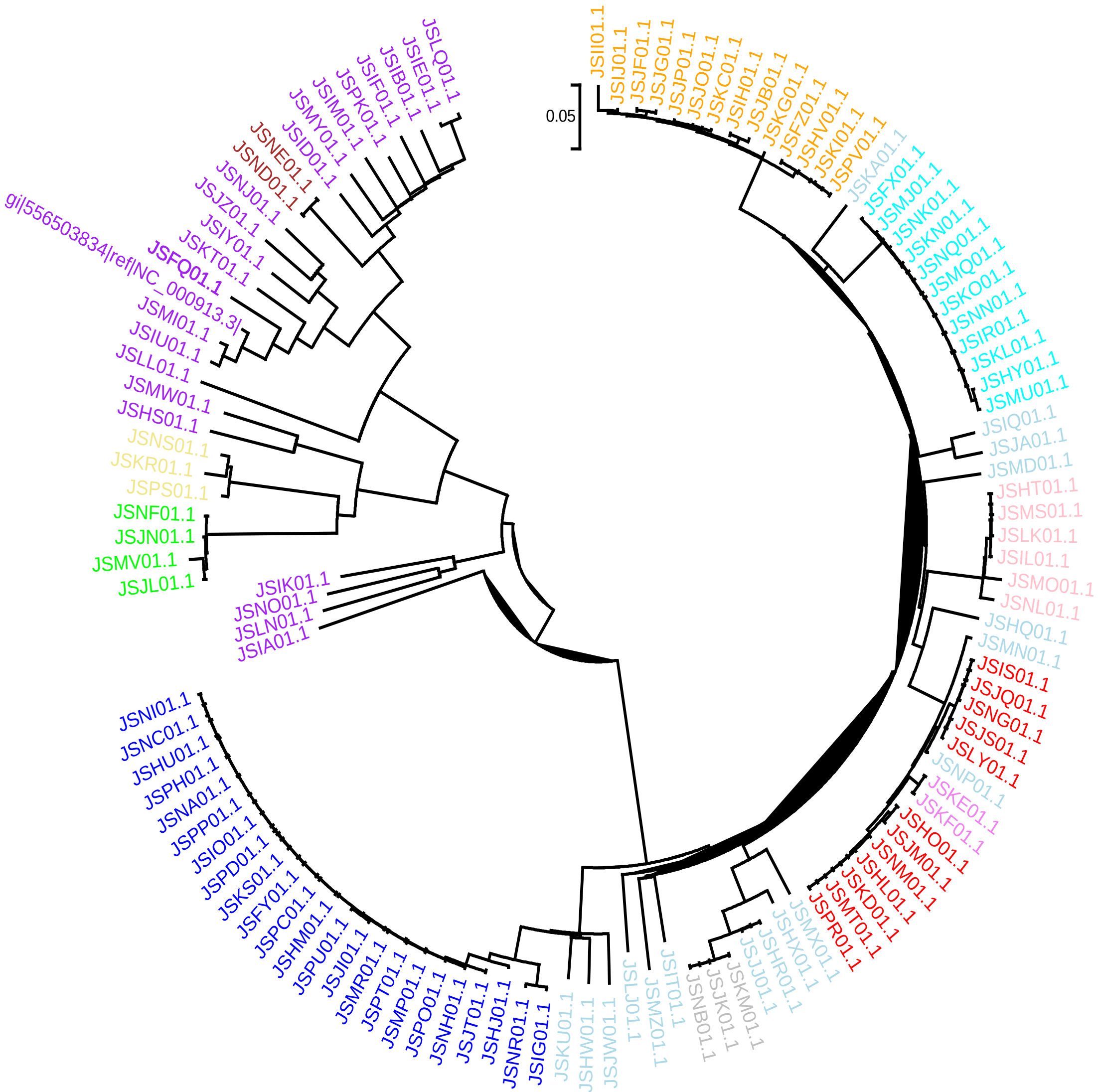

The phylogenetic tree generated from the *E. coli* data. Strains are colored according to the community division presented in Figure 11 in the main text and supplementary Figure S3.
