# Supplementary material for: CONE: Community Oriented Network Estimation Is a Versatile Framework for Inferring Population Structure in Large-Scale Sequencing Data
Source: G3 (Bethesda). 2017 Aug 22;7(10):3359–77. doi: 10.1534/g3.117.300131 (PMC5633386; doi:10.1534/g3.117.300131)
Supplement: Supplementary file 7 [file 3359FigureS7.pdf]

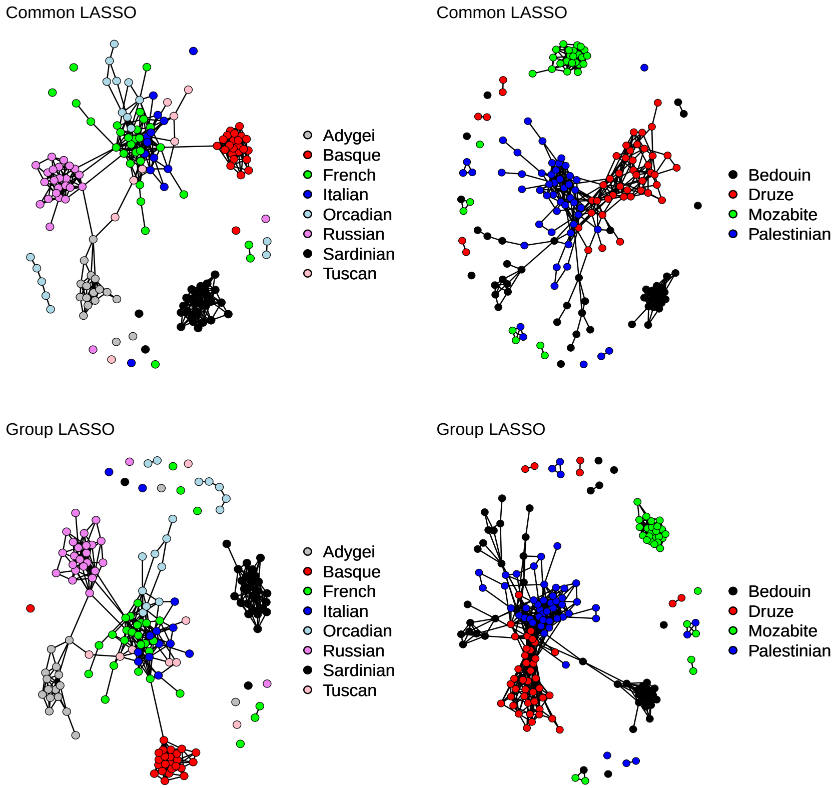

Comparison of the simple LASSO regression with the group LASSO penalty using a small portion of the HGDP data (European and Middle East populations). Networks titled as "Common LASSO" are the same as the Figures 4A and B in the main article. Networks titled as "Group LASSO" are estimated by using the group LASSO penalty in the multinomial LASSO regression in `glmnet` of the R-program.
