# Supplementary material for: CONE: Community Oriented Network Estimation Is a Versatile Framework for Inferring Population Structure in Large-Scale Sequencing Data
Source: G3 (Bethesda). 2017 Aug 22;7(10):3359–77. doi: 10.1534/g3.117.300131 (PMC5633386; doi:10.1534/g3.117.300131)
Supplement: Supplementary file 8 [file 3359FileS1.zip › Supplementary_R_Codes/E_coli/README.rtf]

Before trying to reproduce analyses of the E.Coli data presented in the paper of Kuismin et al (2017), one should recode the data into numerical values 1, 2, 3, 4. Using exactly these numerical values in not necessary but missing values should be handled e.g. with (multiple) imputation.
CONE framework is divided into three different steps and each of them has a distinct R script to make analysis easier(…) to follow:
1) StARSAnalysisEColi.R
Run the StARS procedure to choose the optimal value of the tuning parameter. One will also gain an additional adjacency matrix (or matrices) with element values changing between 0 and 1 representing the strength of the dependency between nodes (samples).  This additional adjacency matrix (named "WeightsWithStARLambdaBactericalData.txt") is saved on the disk.
2) BacterialDataMBStyleNeighborhoodSelection.R
Run the MB-style neighborhood selection using the whole data. One can set the value of the tuning parameter according to the one determined in the previous step.  Once the procedure is completed, the final NOT SYMMETRIC adjacency matrix is saved on the disk ("HGDPMBapproxNonSymmetrix.txt"). One can use either so called “AND” or “OR” rule to make the adjacency matrix symmetric. In Kuismin et al (2017) authors have used the “AND” rule.
Both of the above mentioned files use packages “bigmemory” and “ff” to circumvent the memory limitations of the R program (although not crucial with this data set).
3) DrawGraphEcoliSamples.R
Plot graphs with R package “qgraph” from the adjacency matrices determined in previous steps. In this script, Fruchterman Reingold algorithm is used to divide graph nodes into different communities. 
4) BactericalDataCommunityAnalysis.R
Samples are divided into different communities using the Walktrap algorithm found within the “igraph” package. Fruchterman Reingold algorithm is used to produce interpretable graph output.  Using igraph functions one can easily determine R objects about how samples are divided into different communities. Finally, one can estimate ancestry coefficients. Caution should be taken when computing ancestry estimates with CONE! Although lacking rigid theoretical justification, these estimates can be used to examine of how clearly samples are divided into distinct clusters/communities.
