# Supplementary material for: CONE: Community Oriented Network Estimation Is a Versatile Framework for Inferring Population Structure in Large-Scale Sequencing Data
Source: G3 (Bethesda). 2017 Aug 22;7(10):3359–77. doi: 10.1534/g3.117.300131 (PMC5633386; doi:10.1534/g3.117.300131)
Supplement: Supplementary file 8 [file 3359FileS1.zip › Supplementary_R_Codes/HGDP/README.rtf]

Before trying to reproduce analyses of the HGDP data presented in the paper of Kuismin et al (2017), one should handle missing values e.g. with (multiple) imputation. In the analysis of Kuismin et al (2017) missing genotypes are imputed with the marker mode (once) and one can do it using the R script “HGDPDataRecode”.
This folder contains multiple similar scripts used for CONE analysis in different data subsamples. Their names should be quite self-explanatory:
1) HGDPDataRecode.r
Recode the HGDP data set using R packages ff, ffbase, bigmemory and biganalytics.
2) StARSAnalysisHGDP.R, StARSEuropeanPopulations.R and StARSMiddle-EastPopulations.R
Run the StARS procedure to choose the optimal value of the tuning parameter. One will also gain an additional adjacency matrix (or matrices) with element values changing between 0 and 1 representing the strength of the dependency between nodes (samples).  This additional adjacency matrix (“WeightsWithStARLambda.txt”, “MiddleEastWeightsWithStARLambda.txt”, “EuropeWeightsWithStARLambda.txt”) is saved on the disk.
3) EuropeMBNeighborhoodSelection.R, MiddleEastMBNeighborhoodSelection.R and HGDPMBNeighborhoodSelection.R
Run the MB-style neighborhood selection using samples from European, Middle-East populations or all samples (HGDP) from the HGDP data set. User can set the value of the tuning parameter according to the one determined with the StARS procedure.  Once the neighborhood selection is completed, the final NOT SYMMETRIC adjacency matrix is saved on the disk ("HGDP_Europe_MBapproxNonSymmetrix.tx", “HGDP_MiddleEast_MBapproxNonSymmetrix.txt” and “HGDPMBapproxNonSymmetrix.txt”). One can use either so called “AND” or “OR” rule to make the adjacency matrix symmetric. In Kuismin et al (2017), authors have used the “AND” rule.
4) DrawGraphAllHGDPSamples.R and DrawGraphEuropeAndMidEast.R
Computes and draws graph from the adjacency matrices determined by using the StARS-procedure (an additional graph) and MB-style neighborhood selection. In this script, the “qgraph” package is used to determine the graph.  Fruchterman Reingold algorithm is used to divide graph nodes into different communities.
5) HGDPAncestryCoefficients.R
All HGDP samples are divided into different communities using the Walktrap algorithm found within the “igraph” package. Fruchterman Reingold algorithm is used to produce interpretable graph output.  Using igraph functions one can easily determine R objects about how samples are divided into different communities. Finally, one can estimate ancestry coefficients (see supplementary Figures S5 and S6 in Kuismin et al 2017). Caution should be taken when computing ancestry estimates with CONE! Although lacking rigid theoretical justification, these estimates can be used to examine of how clearly samples are divided into distinct clusters/communities.
6) VanRadenGenomicRelationshipMatrix.R
Compute VanRaden genomic relationship matrix (*) G and save it as “VanRadenGenomicRelationshipMatrix.txt” 
(*) VanRaden, P. M., 2008 Efﬁcient methods to compute genomic predictions. Journal of Dairy Science 91: 4414–4423.
7) GenomicRelationshipsBetweenHGDPIndividuals.R
Van Raden Genomic Relationship matrix is used to identify (genetic)close relatives in the HGDP data. This data is saved into an additional data file “RelatedIndividuals_VanRaden_G_matrix.txt”.
