# Supplementary material for: CONE: Community Oriented Network Estimation Is a Versatile Framework for Inferring Population Structure in Large-Scale Sequencing Data
Source: G3 (Bethesda). 2017 Aug 22;7(10):3359–77. doi: 10.1534/g3.117.300131 (PMC5633386; doi:10.1534/g3.117.300131)
Supplement: Supplementary file 8 [file 3359FileS1.zip › Supplementary_R_Codes/README.rtf]

This directory contains all R codes and instructions needed for reproducing network and community analyses/figures of simulated and empirical data presented in the paper of M. O. Kuismin, J. Ahlinder and M. J. Sillanpää, 2017 CONE: Community oriented network estimation is a versatile framework for inferring population structure in large scale sequencing data. G3: Genes, Genomes, Genetics. (In press). All scripts were developed using RStudio GUI (Version 0.99.892).
Some files and programs, such as EASYPOP program, individual specific HGDP information, HapMap3 genotype data etc. are not found in these directories. We advise to check the original papers and project/author web pages. 
We are willing to help in any problem concerning these files and all files are available upon request.
Each folder contains more detailed README files. 
1) E_Coli
E.coli draft genomes are publically available at NCBI GenBank (http://www.ncbi.nlm.nih.gov/genbank) under accession numbers JSFQ00000000–JSST00000000. The reference strain K12 substrain MG1665 have GenBank ID 556503834.
2) HGDP
All Stanford HGDP SNP Genotyping Data files are deposited at Fondation Jean Dausset-CEPH (http://www.cephb.fr/en/hgdp_panel.php#basedonnees).
3) Teosinte
All SNP genotypes and related information for Zea mays ssp. parviglumis. Z. m. ssp. mexicana and Tripsacum dactyloides files are available from the DRYAD database (DOI:http://dx.doi.org/10.5061/dryad.8m648).
All of these three directories contain R codes which can be used to run each step of the CONE framework:
i) The StARS subsampling analysis
ii) The MB-style neighborhood selection
iii) Community detection methods and network visualization tools 
iv) Tools to estimated ancestry coefficients and plot them with R
4) Simulations
This directory contains two sub-directories:
a) AncestryCoefficientsSimulation: 
Simulate and analyze admixture coefficients
b) PopulationModelSimulation:
Simulate microsatellite data under different models of population structures.
If you have questions or find problems with these files contact Mikko J. Sillanpää (mikko.sillanpaa@oulu.fi) or Markku Kuismin (markku.kuismin@oulu.fi)
