# Supplementary material for: CONE: Community Oriented Network Estimation Is a Versatile Framework for Inferring Population Structure in Large-Scale Sequencing Data
Source: G3 (Bethesda). 2017 Aug 22;7(10):3359–77. doi: 10.1534/g3.117.300131 (PMC5633386; doi:10.1534/g3.117.300131)
Supplement: Supplementary file 8 [file 3359FileS1.zip › Supplementary_R_Codes/Simulations/AncestryCoefficientsSimulation/README.rtf]

These R scripts can be used to reproduce the simulation analysis of admixed population presented in Kuismin et al 2017 - CONE: Community oriented network estimation is a versatile framework for inferring population structure in large scale sequencing data.
1) ModelSelector.txt
An additional R script used to generate columns of the true Q (the ancestry coefficient matrix) matrix independently from symmetric and asymmetric Dirichlet distributions.
2) HapMapSampleDist.r
An additional R script used to generate the allele frequency matrix F from HapMap3 data. In addition, additional analysis were performed using the PLINK software.
3) SimulationFun.R
Compare the accuracy of the ancestry coefficient matrix Q estimates between those estimated with CONE and sNMF. The root mean squared error (RMSE) is used as the accuracy measure. Before running the script, one has to compute the population allele frequency matrix F. A sample of 2500 SNP markers is used.
Predetermined values for the tuning parameter lambda are determined for each Model. The chosen lambda value is determined with the “elbow” method (see script “FindingNmbOfClustersWalkTrap.R”. Note that the value of lambda will depend on the F and Q matrix.
4) FindingNmbOfClustersWalkTrap.R
An arbitrary set of simulated SNP markers is used as a validation set to determine the optimal value of the tuning parameter lambda using the “elbow” method.  This script was originally run side by side with the “SimulationFun” script. Before running script “FindingNmbOfClustersWalkTrap.R” one should use the “SimulationFun” script to: 
i) Sample the SNP markers needed for the simulation analysis.
ii) Simulate the ground truth matrix Q.
iii) Generate a  genotype data set (“SimSnps” data matrix)
Finally, choose the optimal value for the tuning parameter with the elbow method using the “FindingNmbOfClustersWalkTrap.R” by looking at the output plot.
5) SimulationFunSolutionPath.R
Similar to the “SimulationFun” script but now the Q matrix estimated with CONE is compared with the ground truth and the one producing the smallest estimate of the RMSE is chosen. This is done to illustrate comprehensively how accurately CONE can recover admixture coefficients because the tuning parameter selection method may affect the accuracy of the estimated Q matrix.
