# Supplementary material for: CONE: Community Oriented Network Estimation Is a Versatile Framework for Inferring Population Structure in Large-Scale Sequencing Data
Source: G3 (Bethesda). 2017 Aug 22;7(10):3359–77. doi: 10.1534/g3.117.300131 (PMC5633386; doi:10.1534/g3.117.300131)
Supplement: Supplementary file 8 [file 3359FileS1.zip › Supplementary_R_Codes/Simulations/PopulationModelSimulation/README.rtf]

These R scripts can be used to reproduce the simulation analysis of different population structures (estimate the “true” number of populations) in Kuismin et al 2017 - CONE: Community oriented network estimation is a versatile framework for inferring population structure in large scale sequencing data.
Each of the subfolders contains three (3) R scripts and a “Data” folder:
1) FindingNmbOfClustersWalktrap.R
Determine the value of the tuning parameter lambda with the “elbow” method using an arbitrary data set. Of course lambda depends on the data and should be chosen for each data set separately but this is ignored to make the simulations run smoother/faster. Nevertheless, using a fixed value of lambda also demonstrates the consistency of the elbow method.
2) IslandModelSimulationAnalysis.R , HierarchicalIslandModelSimulationAnalysis.R and HierarchicalStepStoneSimulationAnalysis.R 
How accurately sNMF and CONE are able to determine the number of populations for each population structure models: the island model, the hierarchical island model and the hierarchical stepping stone model.
In this process, two different methods are used to determine the value for the tuning parameter:
i) A predetermined value (lambdaModularity) which is determined from an arbitrary validation set
ii) Value determined using the StARS procedure
3) Summary.R
This is an additional file to produce summary statistics over the results.

Place the data simulated with EASYPOP into the “Data” folder. Proposed names for data sets are presented in the EASYPOP syntax files “HierarchicalIslandSyntax.txt”, “HierarchicalStepStoneSyntax.txt” and “IslandModelSyntax.txt”.
