# Supplementary material for: CONE: Community Oriented Network Estimation Is a Versatile Framework for Inferring Population Structure in Large-Scale Sequencing Data
Source: G3 (Bethesda). 2017 Aug 22;7(10):3359–77. doi: 10.1534/g3.117.300131 (PMC5633386; doi:10.1534/g3.117.300131)
Supplement: Supplementary file 8 [file 3359FileS1.zip › Supplementary_R_Codes/Teosinte/README.rtf]

Before trying to reproduce analyses of the Teosinte data presented in the paper of Kuismin et al (2017), one should handle missing values e.g. with (multiple) imputation. In the analysis of Kuismin et al (2017) missing genotypes are imputed with the marker mode (once).
This folder contains multiple similar scripts used for CONE analysis in different data partitions:
1) The whole data set of Pyhäjärvi et al (2013) (“Teosinte” prefix) 
2) Data set containing only samples from the Parviglumis subspecies (“Parviglumis” prefix)
3) Data set containing only samples from the Mexicana subspecies (“Mexicana” prefix)
CONE framework can be divided into three different steps and each of them has a distinct R script to make analysis easier(…) to follow:
1) TeosinteStARSAndMBNeighborhoodSelection.R
i) Runs the StARS procedure to choose the optimal value of the tuning parameter. Procedures in the script will also produce an additional adjacency matrix (or matrices) with element values changing between 0 and 1 representing the strength of the dependency between nodes (samples).  This additional adjacency matrix ("Teosinte_StARSWeights.txt") is saved on the disk.
ii) Performs the MB-style neighborhood selection using the whole data. User has to set the value of the tuning parameter or use the one determined in the previous step.  Once the procedure is completed, the final NOT SYMMETRIC adjacency matrix is saved on the disk ("Teosinte_nonSymmetricMBapprox.txt"). One can use either so called “AND” or “OR” rule to make the adjacency matrix symmetric. In Kuismin et al (2017), authors have always used the “AND” rule.
2) TeosinteDrawGraph.r
Plot graphs with R package “qgraph” from the adjacency matrices determined in previous steps using all Teosinte samples. Fruchterman Reingold algorithm is used to divide graph nodes into different communities.
3) TeosinteFindingNmbOfClustersElbow.R
The number of populations found in the full data set is explored by plotting the network modularity vs. the corresponding number of inferred clusters in the network. This is done by looking an “elbow” in the modularity vs. number of inferred clusters plot. See supplementary Figure S1 in Kuismin et al 2017. 
4) TeosinteAncestryCoefficients.r
Samples are divided into different communities using the Walktrap algorithm found within the “igraph” package. Fruchterman Reingold algorithm is used to produce interpretable graph output.  Using igraph functions one can easily determine R objects about how samples are divided into different communities. Finally, one can estimate ancestry coefficients (see supplementary Figure S2 in Kuismin et al 2017). Caution should be taken when computing ancestry estimates with CONE! Although lacking rigid theoretical justification, these estimates can be used to examine of how clearly samples are divided into distinct clusters/communities.
As mentioned at the beginning of this description, rest of the files can be used to run CONE analysis on both subspecies and to reproduce the original article figure (Figure 9 in Kuismin et al 2017).
